# The spatial–temporal effect of air pollution on individuals’ reported health and its variation by ethnic groups in the United Kingdom: a multilevel longitudinal analysis

**DOI:** 10.1186/s12889-023-15853-y

**Published:** 2023-05-16

**Authors:** Mary Abed Al Ahad, Urška Demšar, Frank Sullivan, Hill Kulu

**Affiliations:** 1 School of Geography and Sustainable Development, University of St Andrews, St Andrews, Scotland, UK; 2 School of Medicine, University of St Andrews, St Andrews, Scotland, UK

**Keywords:** Air pollution, Health, Ethnicity, Spatial–temporal, Longitudinal, United Kingdom

## Abstract

**Background:**

Air pollution is associated with poor health; though it is unclear whether this association is stronger for ethnic minorities compared to the rest of the population. This study uses longitudinal data to investigate the spatial–temporal effect of air pollution on individuals’ reported health and its variation by ethnicity in the United-Kingdom (UK).

**Methods:**

Longitudinal individual-level data from *Understanding Society: the UK Household Longitudinal Study* including 67,982 adult individuals with 404,264 repeated responses over 11 years (2009–2019) were utilized and were linked to yearly concentrations of NO_2_, SO_2_, and particulate-matter (PM10, PM2.5) pollution once at the local authority and once at the census *Lower Super Output Area* (LSOA) of residence for each individual. This allows for analysis at two geographical scales over time. The association between air pollution and individuals’ health (Likert scale: 1–5, Excellent to poor) and its variation by ethnicity was assessed using three-level mixed-effects ordered logistic models. Analysis distinguished between spatial (*between* areas) and temporal (across time *within* each area) effects of air pollution on health.

**Results:**

Higher concentrations of NO_2_, SO_2_, PM10, and PM2.5 pollution were associated with poorer health. Decomposing air pollution into *between* (spatial: across local authorities or LSOAs) and *within* (temporal: across years within each local authority or LSOA) effects showed a significant *between* effect for NO_2_ and SO_2_ pollutants at both geographical scales, while a significant *between* effect for PM10 and PM2.5 was shown only at the LSOAs level. No significant *within* effects were detected at an either geographical level. Indian, Pakistani/Bangladeshi, Black/African/Caribbean and other ethnic groups and non-UK-born individuals reported poorer health with increasing concentrations of NO_2_, SO_2_, PM10, and PM2.5 pollutants in comparison to the British-white and UK-born individuals.

**Conclusion:**

Using longitudinal data on individuals’ health linked with air pollution data at two geographical scales (local authorities and LSOAs), this study supports the presence of a spatial–temporal association between air pollution and poor self-reported health, which is stronger for ethnic minorities and foreign-born individuals in the UK, partly explained by location-specific differences. Air pollution mitigation is necessary to improve individuals’ health, especially for ethnic minorities who are affected the most.

**Supplementary Information:**

The online version contains supplementary material available at 10.1186/s12889-023-15853-y.

## Introduction

Recent global environmental debates have been focused on the issue of air pollution and its impact on human health [1, 2]. Literature has shown an association between air pollution and elevated risks for mortality, clinical prescriptions, doctor visits, and hospital admissions for a range of acute and chronic health conditions, including cancer, cardiovascular and respiratory diseases [2–4]. For example, in Belgium, a 3.5% increase in cardiovascular hospital admissions and a 4.5% increase in ischemic stroke hospital admissions were reported for every 10 μg/m^3^ increase in nitrogen dioxide (NO_2_) pollution [5]. In the United Kingdom (UK), the Committee on the Medical Effects of Air Pollution (COMEAP) has published a series of reports assessing the impacts of long-term exposure to air pollution on health and mortality. In those reports, particulate matter with a diameter ≤ 2.5 µm (PM2.5) was found to be associated with all-cause, cardiopulmonary, and lung cancer mortality [6]. Another stream of the literature revealed the association between increased particulate matter air pollution and poor self-reported health [7–10]. Self-reported health can capture the health status from the perspective of the individual and it is an accurate proxy for objective health measures such as mortality and hospital admissions [11–13].

The impact of air pollution on health is complex and is affected by a number of social, economic, individual, contextual, and environmental factors [2, 4]. Gender, age, poverty, socioeconomic insecurity, educational attainment, marital status, household size and condition, occupation type and level, and income are among the socioeconomic factors affecting the association between air pollution and health [2, 4, 14–16]. Pre-existing comorbidities, individual lifestyle habits (e.g., smoking, exercise, and alcohol consumption), contextual factors (e.g., neighbourhood condition, urbanicity, and population density), and environmental factors (e.g., the season, temperature, relative humidity, rainfall, and wind) also affect the individuals’ exposure to ambient air pollution and its associated illnesses [2, 4, 16–18]. For example, the effect of air pollution on poor self-reported health in France [19] and Germany [20] was exacerbated by increased socioeconomic insecurity, being unemployed, and living in more deprived areas.

Investigating the effect of air pollution on health by key population and socio-demographic subgroups would help in identifying the population sub-groups most affected by air pollution for specific intervention measures. In this context, literature has been focused on examining the effect of air pollution on health by gender, age, education, socioeconomic position, and deprivation. However, research on the effect of air pollution on health by ethnicity and migration status (i.e., being a foreign-born individual) is still lacking in European countries and the UK as per a recently published systematic scoping literature review [4]. Most of the research about the effect of air pollution on health by ethnicity was conducted in the United States of America [7, 21, 22], which is characterized by a different ethnic composition and structure than Europe. To the best of our knowledge, only one study investigated the effect of air pollution on *respiratory-asthma* health in adolescents by ethnic groups in the UK [23]. In this study, Astell-Burt et al. found that despite the higher concentrations of air pollution at the place of residence, ethnic minorities did not show lower lung function than the rest of the population. They even observed a lower prevalence of asthma among some ethnic minority groups compared to the British-white group, even though ethnic minorities lived in more polluted regions [23].

Ethnicity forms an important topic in the health literature [24, 25] and might be an important effect modifier in the association between air pollution and health. Ethnic minorities often report poorer health compared to the rest of the population [8, 25–28]. Literature from the UK has shown that people of Pakistani and Bangladeshi origins tend to have the poorest reported health followed by people from African/Caribbean and Indian origins [8, 25, 29, 30]. This is because ethnic minorities, in general, tend to occupy lower socioeconomic status and live in more deprived ethnic concentration communities with poor housing conditions [25, 31–33]. Racism, inadequate access to healthcare and poor patient service can also explain why some ethnic minority groups in the UK report poorer health [34, 35]. In contrast, foreign-born individuals in the UK tend to have better health and lower rates of mortality compared to the native-born population, which is linked to the “*Healthy migrant effect*” theory [36, 37]. This theory indicates that healthier, more educated, wealthier, and better job market-suited individuals are the ones who have the capability of migrating to high-income countries such as the UK [29, 36].

Thus, in the context of environmental exposures, ethnic minorities in the UK are expected to show poorer health with increased exposure to air pollution due to two main reasons. First, the more disadvantaged socioeconomic status and the experienced racism of this group would increase their risk of illness, making them more sensitive to the health impacts of air pollution. Second, ethnic minorities and foreign-born individuals often reside in large highly populated cities and in low-priced social housing, which is situated in more deprived neighbourhoods and near major roads and industrial areas with little access to green spaces [33]. This increases their exposure to air pollution, mostly traffic-related pollution [38], and results in a stronger effect of air pollution on health. Despite the UK government’s efforts in enhancing the air quality to meet the European Commission guidelines post 2001, an analysis done by Mitchell et al. (2015) found that improvements in air quality were the highest in the least deprived areas, whereas the most deprived areas still suffer from high air pollution in excess of the recommended air quality guidelines [39]. This indicates that ethnic minorities and foreign-born individuals who mostly live in deprived areas will be exposed to higher concentrations of air pollution compared to the rest of the population, which would result in poorer health.

In addition to the lack of studies on the effect of air pollution on health by ethnicity and country of birth, the application of innovative study designs that can differentiate between spatial and temporal components is also lacking. Previous studies have examined the short-term effects of air pollution on health, mortality, and hospital admissions using time series, case-crossover, or ecological designs [3, 4]. The long-term effect of air pollution on health outcomes was also assessed in the literature using cohort designs [3, 4]. However, to our knowledge, no study has used a *between-within* longitudinal design to examine the spatial–temporal effect of long-term exposure to air pollution on health. Applying a *between-within* longitudinal design would involve a decomposition of the air pollution effect on health into *between* (calculating the average air pollution concentration for each geographical area across the follow-up time) and *within* (calculating the annual deviation in air pollution concentrations from the area-average *between* concentration for each unit of time within the follow-up period) effects [40]. This approach would allow us to distinguish between the *spatial (between)* and the *temporal (within)* effects of air pollution on individuals’ health.

Finally, studies that link air pollution data to individual-level data at different geographical scales are lacking. Assessing the effect of air pollution on individuals’ health at two geographical scales (e.g., coarse local authorities and detailed census areas such as lower super output areas (LSOAs)) would allow for a comparison of the results between the two scales and a detailed exploration of the local-contextual patterns. Whilst analysis at a finer geographical scale using LSOAs would add spatial robustness to the results, analysis at a coarser scale using local authorities would better inform local mitigation approaches by providing overall area estimates for the local authority boards. Additionally, daily exposure to air pollution does not occur only at the place of residence. Thus, assessing the exposure at a coarser geographical scale such as at the local authority may capture exposures at the workplace and during commuting, if the individual lives, works, and does most of the daily activities within the same local authority. This means that coarser local authorities are a better proxy for exposure to risk on a day-to-day basis compared to the finer LSOAs.

In this study, we will be using two geographical scales (coarse local authorities and detailed LSOAs) to assess the spatial–temporal (*between-within*) effects of air pollution on individuals’ reported health and how this effect varies by ethnicity and country of birth (being a foreign-born versus not) in the UK. The UK is composed of four nations: England, Wales, Scotland, and Northern Ireland. Each of these four nations has its own classification of census areas. In England and Wales, the most geographically detailed census areas are output areas with LSOAs being an aggregation of output areas that are used to decompose England and Wales based on the population size into areas with a minimum population size of 1000 people. Those are equivalent to data zones in Scotland and to Super Output Areas in Northern Ireland. For simplicity, we will refer in this article to the joint LSOAs, data zones, and Super Output Areas as LSOAs.

### Study objectives

The objectives of this study are as follows:

1. To investigate the association between long-term (11 years) exposure to NO_2_, sulphur dioxide (SO_2_), Particulate matter with diameter ≤ 10 µm (PM10), and PM2.5 air pollutants and individuals’ reported health.
2. To investigate the *between* (spatial—average pollutant concentration across the follow-up time for each geographical area) and *within* (temporal—annual deviation in the pollutant concentration from the average area concentration for each time unit of the follow-up period) effects of air pollution on individuals’ reported health.
3. To examine how the association of air pollution with individuals’ reported health varies by different ethnic groups and migration status (being a foreign-born versus UK-born individual).

The analysis to meet the above objectives will be performed on two datasets: first on the dataset which includes the linkage of air pollution to the individual-level data at the LSOAs level, and second on the dataset which includes the linkage of air pollution to the individual-level data at the local authority level.

## Methods

### Study design and population

A longitudinal panel design was employed using individual-level data from “*Understanding Society: The UK Household Longitudinal Study*” [41]. Understanding Society is a rich longitudinal dataset consisting of 10 data collection waves/panels that span from 2009 up to 2020 with around 40,000 households recruited at wave 1 from the four nations of the UK: England, Wales, Scotland, and Northern Ireland. It involves two main surveys: the youth survey which is filled out by young people (aged 10 to 15) and the adult survey which is filled by individuals aged 16 and above [41].

The dataset includes information on the socio-demographic characteristics of individuals (e.g., age, gender, marital status, educational attainment, occupation, housing tenure, perceived financial situation, ethnicity, and country of birth) and on the individuals’ self-reported health, well-being, smoking status, as well as the local authority/council area and census Lower Super Output Areas (LSOAs) where households are located. Individuals recruited in the Understanding Society study are visited each year to collect information on changes to their household and individual circumstances [41].

The sample design of the Understanding Society main survey is made up of four components: 1) the large General Population Sample (around 26,000 recruited households at wave 1, 2009–2010); 2) the Ethnic Minority Boost Sample (around 4000 recruited households at wave 1, 2009–2010); 3) the former British Household Panel Survey sample (at wave 1, 2010); and 4) the Immigrant and Ethnic Minority Boost Sample (around 2,500 recruited households at wave 6, 2015) [41]. Further information on the Understanding Society study design is described elsewhere [42, 43].

**Fig. 1:**
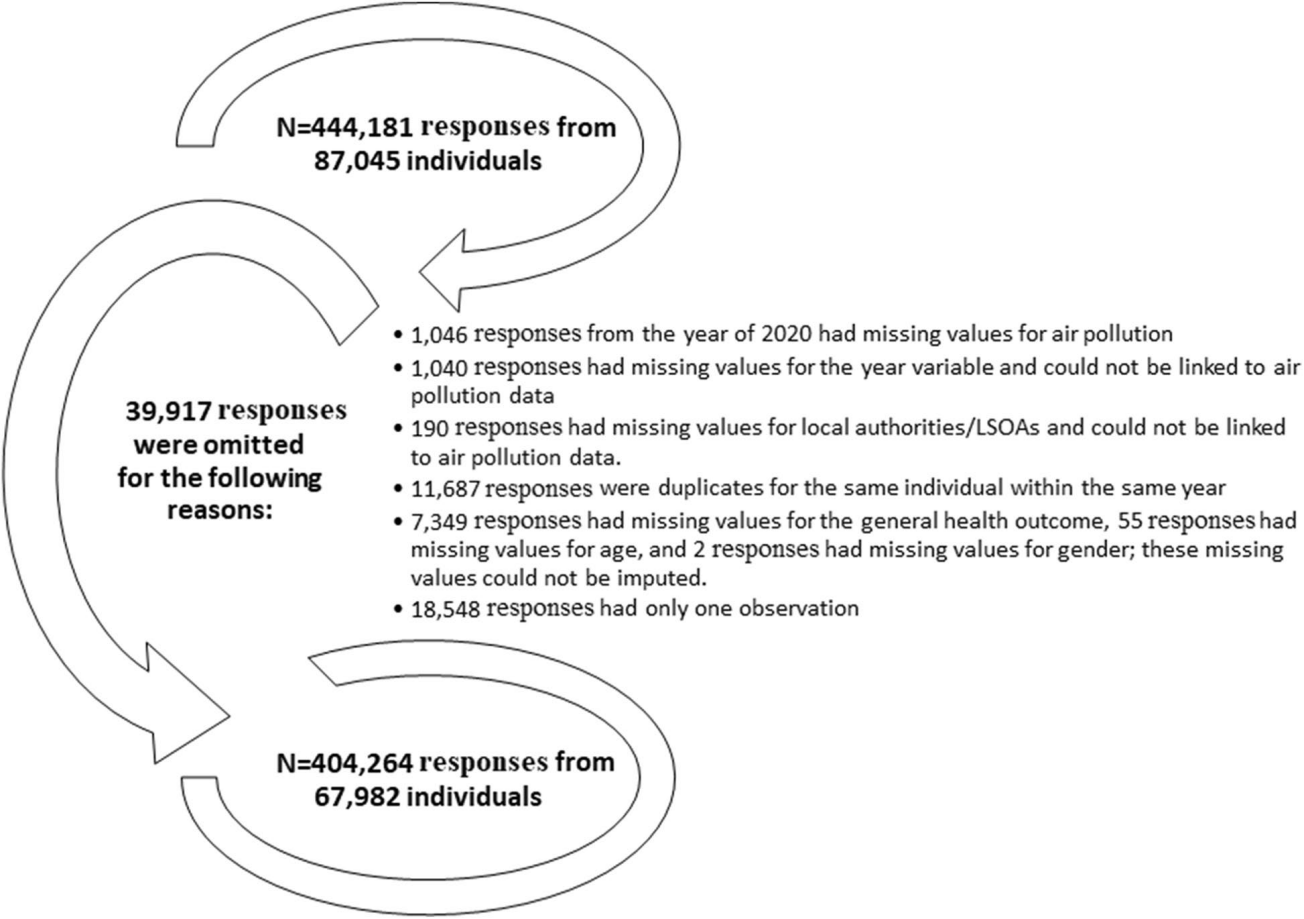
The reasons for omitting survey responses from the UK household longitudinal data

For this study, we utilized individual-level data on 67,982 individuals with 404,264 repeated responses (at least 2 repeated responses per individual) across 10 data collection waves over 11 years (2009–2019) from the adult survey (age: 16 +) of the Understanding Society data. It is worth noting that the initial adult survey of the Understanding Society data involved a total of 87,045 individuals with 444,181 repeated responses and that 39,917 observations were deleted due to the reasons summarised in Fig. 1.

### Variables and measurements

#### Self-reported health

Individuals’ self-reported health which asks how individuals perceive their health in general is assessed on a 5-point Likert scale: 1 = excellent, 2 = very good, 3 = good, 4 = fair, 5 = poor. Out of the total 404,264 general health observations, 105 (0.03%) were missing and were filled out using another health indicator: satisfaction with health, which showed a strong correlation (Pearson’s coefficient = 0.53) with the general health outcome. Satisfaction with health is measured on a 7-point Likert scale (completely satisfied, mostly satisfied, somewhat satisfied, neither satisfied nor dissatisfied, somewhat dissatisfied, mostly dissatisfied, and completely dissatisfied). Therefore, completely satisfied was coded to excellent health, mostly satisfied was coded to very good health, somewhat satisfied was coded to good health, neither satisfied nor dissatisfied and somewhat dissatisfied were coded to fair health, and mostly dissatisfied and completely dissatisfied were coded to poor health.

It should be noted that individuals’ self-reported health was chosen as the main outcome in this study due to its ability to capture the health status from the perspective of the individual and it is considered a reliable measure of health given the high observed correlations between self-reported health and objective health measures (e.g., mortality and hospital admissions) in the literature [11–13].

#### Air pollution

We obtained yearly air pollution data that combine all sources of air pollution including road traffic and industrial/combustion processes for NO_2_, SO_2_, PM10, and PM2.5 pollutants from the “Department for Environment Food and Rural Affairs” online database [44]. These are raster data of mean annual concentrations of pollutants measured in µg/m^3^ up to the year 2019, estimated using air dispersion models at a spatial resolution of 1 × 1 km^2^, and projected using the UK National Grid [44]. The raster data is projected in a way that each 1 × 1 km^2^ raster square has the value of a central air pollution point.

For each of the 391 local authorities/council areas in the UK, we computed the average concentration of NO_2_, SO_2_, PM10, and PM2.5 pollution from all the centroids of the 1 × 1 km^2^ raster cells that intersected/fell within the boundaries of the respective local authorities/council areas for each year from 2009 up to 2019. These average concentrations of air pollution were then linked to the “Understanding Society” data using the individuals’ local authority of residence for each year of observation per individual between 2009 and 2019, inclusive.

To minimize exposure bias and establish more robust results, we also linked the 1 × 1 km^2^ raster air pollution data to the Understanding Society data at the level of Lower Super Output Areas (LSOAs; data zones for Scotland and Super Output Areas for Northern Ireland), a finer geographical scale, for each individual and each year of follow-up (2009–2019). The linkage was done by calculating an area-weighted average air pollution concentration for each LSOA based on the proportion of area intersection between the 1 × 1 km^2^ raster squares and the respective LSOA. For example, if a LSOA intersected with three 1 × 1 km^2^ squares in which one intersection covered half of the area of that LSOA while the other two intersections covered a proportion of 0.3 and 0.2, respectively; the air pollution concentration for that LSOA would be 0.5 × air pollution concentration of the first intersected square + 0.3 × air pollution concentration of the second intersected square + 0.2 × air pollution concentration of the third intersected square. Using these smaller spatial units, we conducted our analysis at a smaller geographic scale than local authorities, which allowed us to explore local-contextual patterns of the effect of air pollution on health.

**Fig. 2:**
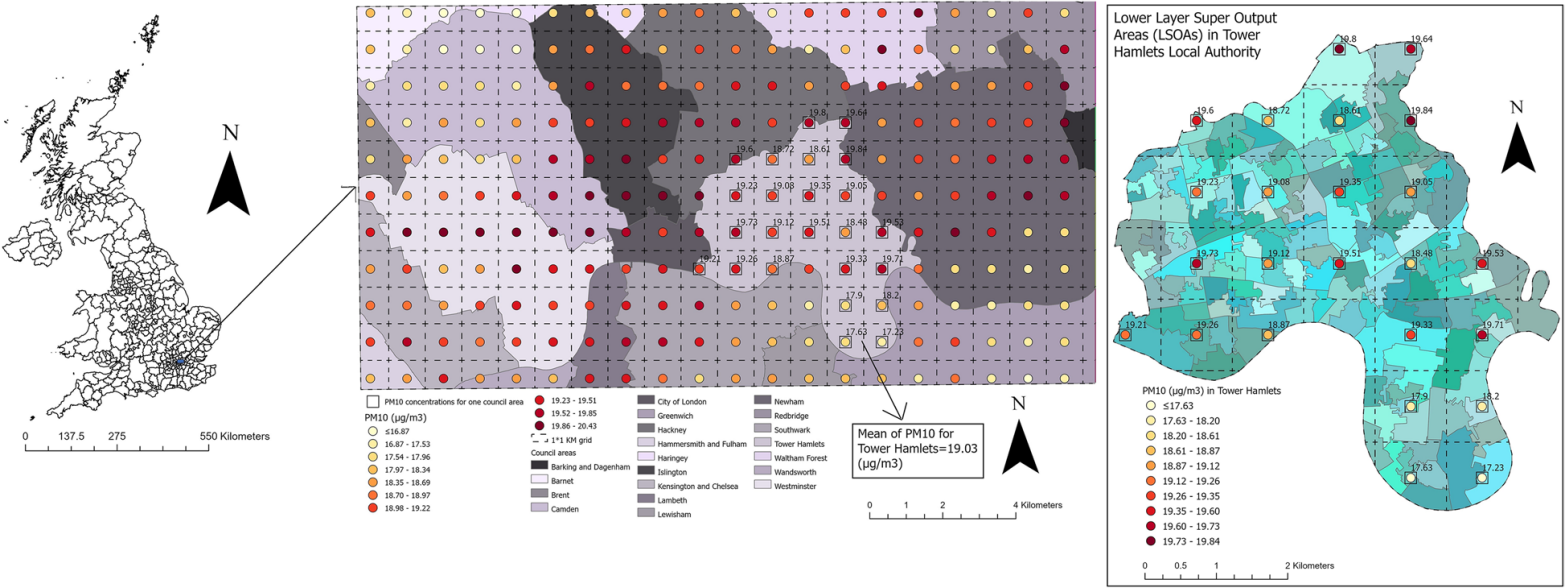
A map showing the local authorities in the UK and an enlarged subset of 20 local authorities in the south-east of the UK with an example of PM10 concentrations at 1 × 1 km^2^ grid for the year 2017 for Tower Hamlets local authority and its corresponding LSOAs. The green–blue coloured polygons in the LSOAs map represent the LSOAs; The map was constructed by the authors in ArcGIS Pro software using PM10 air pollution shapefile for the year of 2017 downloaded from the DEFRA online data repository [44], local authorities UK boundaries shapefile downloaded from the Office for National Statistics [45], and LSOAs and data zones UK boundaries also downloaded from the Office for National Statistics, National Records of Scotland, and Northern Ireland Statistics [46]. Both DEFRA and Office for National Statistics shapefiles are governed under the Open Government Licence v.3.0

A map showing the local authorities in the UK (council areas in Scotland) and an enlarged subset of 20 local authorities in the southeast of the UK with an example of PM10 concentrations at 1 × 1 km^2^ grid for the year 2017 for Tower Hamlets local authority and its corresponding LSOAs was used to illustrate the process of air pollution linkages (Fig. 2).

#### Socio-demographic and lifestyle covariates

In this study, ethnicity (Other-white, Pakistani/Bangladeshi, Indian, Black/African/Caribbean, mixed ethnicities, and other ethnicities versus British-white (Reference category)) and country of birth (non-UK-born and missing information versus UK-born (Reference category)) covariates were considered as effect modifiers in the association between air pollution and self-reported health.

Additionally, we selected a list of individual-level socio-demographic and lifestyle covariates based on what is available in the Understanding Society data and based on the confounders considered by the air pollution-health literature [3, 4]. These included age (coded as 16–18 and then in 5 years increments as 19–23; 24–28; 29–33; 34–38; 39–43; 44–48; 49–53; 54–58; 59–63; 59–63; 64–68; 69–73; 74–78; > 78); gender (females versus males (Reference category)); marital status (living as a couple, single never married, divorced/separated, widowed, and missing information versus married (Reference category)); educational attainment (High school, lower education, other educational qualifications, and still a student versus university degree (Reference category)); occupation (Non-manual workers, manual workers, student/retired/not-working and missing information versus managers/professionals/ employers (Reference category)); housing tenure (Owned with mortgage, local authority rent, housing association rent, private rent and other or missing information versus owned outright (Reference category)); perceived financial situation (living difficultly and missing information versus living comfortably/doing alright (Reference category)); and smoking (smoker and missing information versus non-smoker (Reference category)) [47].

The question about smoking was not asked during wave 1 of data collection and during waves 3 and 4 for people above the age of 21 years old. Therefore, individual responses on smoking from wave 2 were used as a proxy for the smoking status in waves 1, 3, and 4 [47]. This imputation is unlikely to deviate from the real smoking status scenario because the intraclass correlation coefficient (ICC) indicates a 97% similarity in the individual smoking responses across the data collection waves.

Finally, year dummies (calendar year: 2009–2019) were considered as a control for the time trend in our analysis following the approach of relevant studies [48, 49]. Given that our study utilises yearly air pollution data, controlling for other temporal covariates considered by relevant literature such as seasonal trends [50, 51] was not possible.

### Data analysis

Percentages were computed to describe the individuals’ socio-demographic and lifestyle factors for each wave (waves 1 to 10) of the Understanding Society sample. We also examined the correlation between NO_2_, SO_2_, PM10, and PM2.5 pollutants at the two geographical scales of local authorities and LSOAs using Pearson’s correlation coefficient. Given the high observed correlations between the pollutants (Pearson’s coefficient ≥ 0.7 [52]; Tables 2 and 3), the association of NO_2_, SO_2_, PM10, and PM2.5 pollutants with self-reported health was examined in separate regression models. However, a low to moderate correlation was observed between SO_2_ and each of the other three pollutants, which enabled the construction of bi-pollutant models adjusting the NO_2_, PM10, and PM2.5 models for the SO_2_ pollutant.

Intraclass correlation coefficients (ICCs) were computed to assess the homogeneity in the self-reported general health responses within individuals and household clusters. An ICC of more than 0.3 indicates the presence of fair to high homogeneity in the responses within the examined clusters across time [53]. Given the presence of 65% homogeneity (ICC = 0.65; Table 4) within the responses of self-reported health for each individual across time, the mean of self-reported health was calculated from predictions of mixed-effects linear models, which were adjusted for age in fixed effects and for the individual ID in the random intercept.

Three-level (repeated individual observations across time nested within local authorities or LSOAs) mixed-effects ordered logistic models were used to assess the association between self-reported general health and each of NO_2_, SO_2_, PM10, and PM2.5 pollutants. Mixed-effects ordered logistic models were used to account for the nested-longitudinal structure of the data and because general health is an ordinal outcome, which is measured on a 5-point (Excellent, very good, good, fair, and poor health) Likert scale. These models were adjusted for the socio-demographic and lifestyle covariates and the year (2009–2019) dummies. The models which involve air pollution linked at the LSOAs level were additionally adjusted for the LSOAs population density. This was done to account for any bias introduced by the LSOAs being constructed by dividing areas in the four nations of the UK based on the population size. In a supplementary analysis, we also demonstrate the association of self-reported health with each of the socio-demographic and lifestyle covariates (Additional file 1: Supplementary Table 1). It is worth noting that we did not account for the household clustering in the random intercept of the mixed-effects ordered logistic models due to the low observed homogeneity in the self-reported health responses within each household cluster (ICC = 0.24; Table 4).

In further analysis, we decomposed the overall effect of air pollution (linked at the local authority or LSOAs level) on health into *between* (*spatial*) and *within* (*temporal*) effects. *Between* effects (Eq. 1) were used to determine the *spatial* effect of air pollution by computing the mean of air pollution across the 11 years of follow-up (2009–2019) for each local authority and each LSOA. On the contrary, *within* effects (Eq. 2) were used to determine the *temporal* effect of air pollution by calculating the yearly air pollution deviation from the 11 years mean for each local authority and LSOA. The multilevel mixed-effects ordered logistic models were used to examine the overall (Eq. 3) effect of air pollution as well as the *between* and *within* effects (Eq. 4) of air pollution on self-reported health at two geographical scales (coarse local authorities and detailed LSOAs).

Finally, we incorporated into the mixed-effects models an interaction term between ethnicity and each of NO_2_, SO_2_, PM10, and PM2.5 pollutants and between country of birth and each of the four pollutants to assess whether the association between air pollution and health varies between ethnic groups and by country of birth. Interaction terms were incorporated into the overall pollutant models (Eqs. 5 and 6) and into the *between-within* models, each at a time. Coefficient plots were used to visualize the interaction analysis results.

1 $${\text{Between pollutant concentration}}_{\text{tij}}=\overline{{\text{overall pollutant concentration} }_{\text{j}}}$$

2 $${\text{Within pollutant concentration}}_{\text{tij}}={|\text{overall pollutant concentration}}_{\text{tj}}-\overline{{\text{ overall pollutant concentration} }_{\text{j}}}|.$$

Where i is the individual; t is the time in years; and j is the local authority or LSOA.

3 $$\mathit{ln}\left(\frac{{Y}_{\mathit{ctij}}}{1-{Y}_{\mathit{ctij}}}\right)={\beta }_{c} + {U}_{cij}+{U}_{cj} + {\beta 1\text{overall pollutant concentration}}_{tij}+ {\beta 2\text{Age}}_{tij}+ {\beta 3\text{Gender}}_{tij}+ {\beta 4\text{Ethnicity}}_{tij}+ {\beta 5\text{Country of birth}}_{tij}+ {\beta 6\text{Marital status}}_{tij}+ {\beta 7\text{Education}}_{tij}+{\beta 8\text{Occupation}}_{tij}+{\beta 9\text{Housing tenure}}_{tij}+ {\beta 10\text{Perceivedfinancial situation}}_{tij}+ {\beta 11\text{Smoking status}}_{tij}+ {\beta 12\text{Year dummies}}_{ij}+ {\varepsilon }_{tij}$$

4 $$\mathit{ln}\left(\frac{{Y}_{\mathit{ctij}}}{1-{Y}_{\mathit{ctij}}}\right)={\beta }_{c} + {U}_{cij}+{U}_{cj} + {\beta 1\text{Between pollutant concentration}}_{tij}+ {\beta 2\text{Within pollutant concentration}}_{tij}+ {\beta 3\text{Age}}_{tij}+ {\beta 4\text{Gender}}_{tij}+ {\beta 5\text{Ethnicity}}_{tij}+ {\beta 6\text{Country of birth}}_{tij}+ {\beta 7\text{Marital status}}_{tij}+ {\beta 8\text{Education}}_{tij}+ {\beta 9\text{Occupation}}_{tij}+ {\beta 10\text{Housing tenure}}_{tij}+ {\beta 11\text{Perceived financial situation}}_{tij}+ {\beta 12\text{Smoking status}}_{tij}+ {\beta 13\text{Year dummies}}_{ij} + {\varepsilon }_{tij}$$

5 $$\mathit{ln}\left(\frac{{Y}_{\mathit{ctij}}}{1-{Y}_{\mathit{ctij}}}\right)={\beta }_{c} + {U}_{cij}+{U}_{cj} + \beta 1\text{overall pollutant concentration}\times {\text{Ethnicity}}_{tij} + {\beta 4\text{Age}}_{tij}+ {\beta 5\text{Gender}}_{tij}+ {\beta 6\text{Country of birth}}_{tij}+ {\beta 7\text{Marital status}}_{tij}+ {\beta 8\text{Education}}_{tij}+ {\beta 9\text{Occupation}}_{tij}+ {\beta 10\text{Housing tenure}}_{tij}+ {\beta 11\text{Perceivedfinancial situation}}_{tij}+ {\beta 12\mathrm{Smoking status}}_{tij}+ {\beta 13\mathrm{Year dummies}}_{ij} + {\varepsilon }_{tij}$$

6 $$\mathit{ln}\left(\frac{{Y}_{\mathit{ctij}}}{1-{Y}_{\mathit{ctij}}}\right)={\beta }_{c} + {U}_{cij}+{U}_{cj} + \beta 1\text{overall pollutant concentration}\times {\text{Country of birth}}_{tij} + {\beta 4\text{Age}}_{tij}+ {\beta 5\text{Gender}}_{tij}+ {\beta 6\text{Ethnicity}}_{tij}+ {\beta 7\text{Marital status}}_{tij}+ {\beta 8\text{Education}}_{tij}+ {\beta 9\text{Occupation}}_{tij}+ {{\beta 10\text{Housing tenure}}_{tij}+ \beta 11\text{Perceivedfinancial situation}}_{tij}+ {\beta 12\text{Smoking status}}_{tij}+ {\beta 13\text{Year dummies}}_{ij} + {\varepsilon }_{tij}$$

where *Y*_*ctij*_ is the health outcome for individual *i* measured using 5 ordered categories (c = 1, 2, 3, 4, 5), in local authority or LSOA *j* at year *t*; *β*_*1*_, *β*_*2*_ …. *β*_*12*_ are the slopes of fixed effects; *β*_*c*_ is the fixed intercept for the 5 ordered categories (c = 1, 2, 3, 4, 5); *U*_*cij*_ is level 2 random intercept of individuals nested in local authorities or LSOAs for the 5 ordered categories (c = 1, 2, 3, 4, 5); *U*_*cj*_ is level 3 random intercept of local authorities or LSOAs for the 5 ordered categories (c = 1, 2, 3, 4, 5); *ε*_*tij*_ are the model residuals; Models involving air pollution linked at the LSOAs level are additionally adjusted for the LSOAs population density.

In a sensitivity analysis, we performed the same multilevel mixed-effects ordered logistic models to examine the overall and the *between-within* effects of air pollution (linked at the level of local authority and LSOAs) on self-reported health and how these effects vary by ethnic groups and country of birth only for individuals recruited in wave 1 of the Understanding Society data. This sensitivity analysis was carried out to balance the cohort effect because not all individuals in our sample were recruited in wave 1 and attrition bias is more probable at later waves.

In a sensitivity analysis, we also carried out four-level mixed-effects logistic models with repeated individual responses nested in LSOAs, nested in local authorities to examine the association between air pollution linked at the LSOAs level and self-reported general health coded as a binary variable of fair/poor health versus excellent/very good/good health. This sensitivity analysis was conducted to assess in more details the local and regional effects of air pollution on health in the general population and by ethnicity. Given the complexity of the four-level nested models, the analysis was performed using a binary version of the general health outcome rather than the ordered Likert scale version.

Statistical analysis was conducted using STATA software (StataCorp. 2015. Stata Statistical Software: Release 14. College Station, TX: StataCorp LP) and spatial pre-processing was conducted using ArcGIS Pro software. Regression results were reported in terms of odds ratios (ORs) and 95% confidence intervals (CIs) per 10 µg/m^3^ increase in air pollution. Statistical significance was considered at a *P*-value of less than 0.05.

## Results

### Description of individuals’ socio-demographic and lifestyle factors

This study included a total of 67,982 adult individuals (aged 16 +) with 404,264 repeated responses across 10 waves/panels spread over 11 years (2009–2019) of individuals’ follow-up. The average number of observations per individual was 5.95 (SD = 2.86) with a minimum of 2 observations per individual and the mean follow-up time was 5.53 (SD = 3.00) years.

Table 1 summarises the descriptive statistics for the individuals’ socio-demographic and lifestyle factors for each of the 10 waves/panels of the Understanding Society sample. In all the waves, most of the individuals were females, aged between 34 and 58 years old, were married, were non-manual workers (if working), owned their houses either outrightly or with a mortgage, had a comfortable/alright financial situation, were non-smokers, and lived in England. As for educational attainment, individuals were equally distributed between high school, university, and other educational qualifications in all the waves (Table 1).

**Table 1:** Description of individual’s socio-demographic and lifestyle factors for each wave of the Understanding Society data (*N* = 404,264 observations from 67,982 individuals)

		**Wave1 (2009–2011)**	**Wave2 (2010–2012)**	**Wave3 (2011–2013)**	**Wave4 (2012–2014)**	**Wave5 (2013–2015)**	**Wave6 (2014–2016)**	**Wave7 (2015–2017)**	**Wave8 (2016–2018)**	**Wave9 (2017–2019)**	**Wave10 (2018–2019)**
		*N* = 39,798	*N* = 50,728	*N* = 47,651	*N* = 45,236	*N* = 42,525	*N* = 39,777	*N* = 38,518	*N* = 36,264	*N* = 33,141	*N* = 30,626
		**Percent**	**Percent**	**Percent**	**Percent**	**Percent**	**Percent**	**Percent**	**Percent**	**Percent**	**Percent**
**Gender**	Male	45	45	46	46	46	46	46	46	45	45
	Female	56	55	54	54	54	54	54	55	55	55
**Age**	Young (< 34)	27	27	26	26	26	26	25	24	23	21
	Middle age (34–58)	46	44	44	44	43	44	44	44	43	43
	Old (> 58)	27	29	30	30	31	30	31	33	34	35
**Ethnicity**	British-white	76	77	78	78	78	74	74	75	77	78
	Other-white	4	5	5	5	4	6	5	5	5	5
	Indian	4	3	3	3	3	4	4	4	4	4
	Pakistani/Bangladeshi	5	4	4	4	4	5	5	5	5	5
	Black/African/Caribbean	5	4	4	4	4	4	4	4	4	3
	Mixed ethnicities	2	1	1	2	2	2	2	2	2	2
	Other ethnicities	4	5	5	5	5	5	5	5	4	3
**Country of birth**	Born in the UK	83	67	67	67	68	67	67	67	68	69
	Not born in the UK	17	14	13	13	12	15	15	15	13	12
	Missing information	0	20	21	20	20	18	18	18	19	19
**Marital status**	Married	53	53	53	52	52	53	53	53	54	55
	Living as a couple	11	11	11	11	11	11	11	10	10	10
	Widowed	6	6	6	6	6	6	6	6	6	6
	Divorced/separated	9	8	8	8	8	8	8	8	8	8
	Single never married	21	22	22	22	23	23	24	23	23	21
	Missing information	0	0	0	0	0	0	0	0	0	0
**Education**	University degree	30	25	26	27	28	29	30	31	32	34
	High school degree	32	26	26	27	27	27	27	27	27	27
	Lower educational levels	1	1	1	1	1	1	1	1	1	1
	Other qualifications	30	40	40	38	37	36	35	35	34	33
	Still a student	7	7	7	7	7	7	7	6	6	5
**Occupation**	Managers/Professionals/employers	12	12	12	12	12	12	12	12	12	12
	Non-manual workers	26	26	26	26	26	27	27	27	26	26
	Manual workers	18	18	18	18	19	19	19	18	17	17
	Not applicable: Student/ retired/Not working	44	44	44	43	43	41	41	42	42	43
	Missing information	0	0	1	1	0	1	1	1	2	3
**Housing tenure**	Owned outright	29	31	31	32	33	33	34	34	35	36
	Owned with mortgage	39	39	39	39	38	37	37	37	37	37
	Local authority rent	11	11	10	10	10	9	9	9	8	8
	Housing association rent	7	7	7	7	7	7	7	7	6	6
	Private rent	13	12	12	11	11	12	12	11	10	10
	Other or missing information	1	0	0	1	2	2	1	2	3	3
**Perceived financial situation**	living comfortably/doing alright	56	57	57	59	60	65	67	70	69	70
	living difficultly	40	37	36	33	32	27	26	26	27	28
	Missing information	4	6	8	8	8	8	7	5	3	3
**Smoking status**	non-smoker	70	74	69	68	75	71	79	82	84	85
	smoker	19	20	18	18	16	15	15	14	13	13
	Missing information	11	6	13	14	8	14	7	4	3	2
**Nation**	England	84	77	76	77	77	79	79	79	78	79
	Wales	5	8	8	8	7	7	6	7	7	7
	Scotland	7	9	9	9	9	8	8	8	9	9
	Northern Ireland	4	7	7	7	6	6	6	6	7	6

Most of the individuals were born in the UK (83% in wave 1) and belonged to British-white ethnicity (76% in wave 1). The share of ethnic minorities in wave 1 was as follows: Other-white (4%), Indians (4%), Pakistani/Bangladeshi (5%), Black/African/Caribbean (5%), mixed ethnicities (2%), and other ethnicities (4%) (Table 1).

### Description of air pollution

#### Description of air pollution at the LSOAs level

**Fig. 3:**
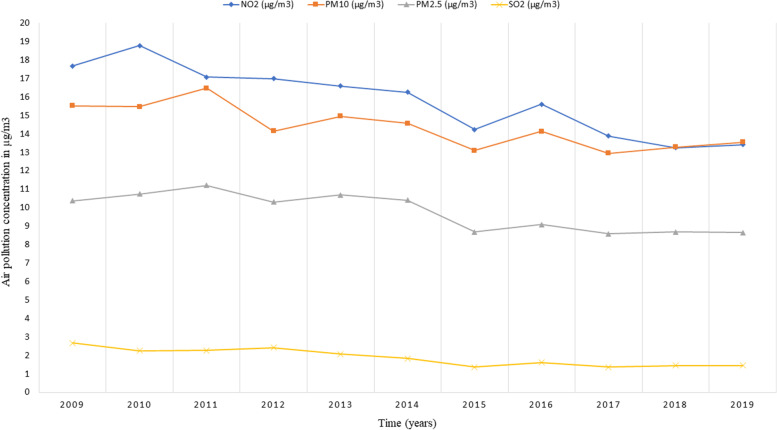
The annual mean of NO_2_, SO_2_, PM10, and PM2.5 air pollutants at the LSOAs level in the UK from the year 2009 to 2019 (*N* = 42,619 LSOAs)

The mean of NO_2_, SO_2_, PM10, and PM2.5 pollutants across 42,619 LSOAs in the UK for each year from 2009 through 2019 is summarised in Fig. 3. Fluctuations in air pollution were observed from one year to another with lower levels of pollution noted in the last 5 years of the observation window in comparison to previous years for all four pollutants, with an exception for the year 2016 (Fig. 3). The yearly concentrations of NO_2_ decreased from 17.6 µg/m^3^ in 2009 to 13.4 µg/m^3^ in 2019. Similarly, the concentrations of PM10 and PM2.5 decreased from 15.5 µg/m^3^ in 2009 to 13.5 µg/m^3^ in 2019 and from 10.4 µg/m^3^ in 2009 to 8.6 µg/m^3^ in 2019, respectively. A decline in SO_2_ concentration from 2.7 µg/m^3^ in 2009 to 1.4 µg/m^3^ in 2019 was also observed (Fig. 3).

Table 2 summarises the average concentrations of air pollutants across 42,619 LSOAs in the UK and their respective correlations. A high correlation (Pearson’s coefficient ≥ 0.7) was noted between NO_2_, PM10, and PM2.5 pollutants, which could be explained by the chemical reactions between particulate matter and NO_2_ pollutants in the atmosphere (Table 2).

**Table 2:** Exposure description and correlation matrix of air pollutants at the LSOAs level (*N* = 42,619 LSOAs)

	Pearson’s correlation coefficient							
	NO_2_ (µg/m^3^)	SO_2_ (µg/m^3^)	PM10 (µg/m^3^)	PM2.5 (µg/m^3^)	Mean	SD	Median	Interquartile range
NO_2_ (µg/m^3^)	1.00				15.80	7.47	14.91	9.69
SO_2_ (µg/m^3^)	0.36	1.00			1.89	1.26	1.59	1.06
PM10 (µg/m^3^)	**0.76**	0.28	1.00		14.38	3.18	14.53	4.32
PM2.5 (µg/m^3^)	**0.79**	0.31	**0.97**	1.00	9.76	2.40	9.88	3.30

Strong correlations with a correlation coefficient ≥ 0.70 are highlighted in bold

#### Description of air pollution at the Local authority level

**Fig. 4:**
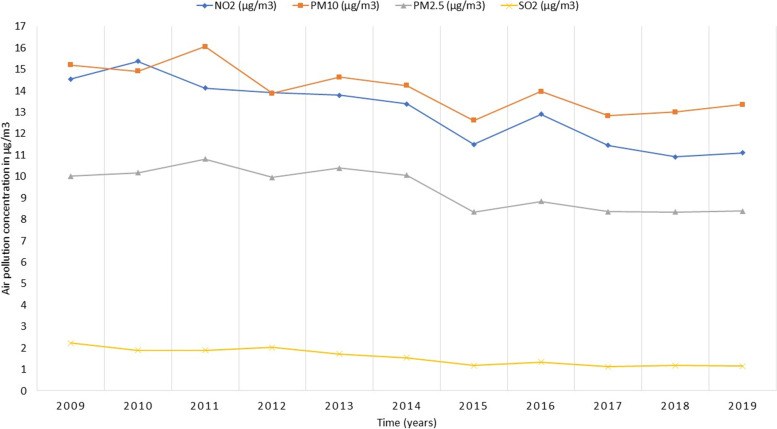
The annual mean of NO_2_, SO_2_, PM10, and PM2.5 air pollutants at the local authority level in the UK from the year 2009 to 2019 (*N* = 391 local authorities)

Similar to the LSOAs, fluctuations in the mean of NO_2_, SO_2_, PM10, and PM2.5 concentrations across 391 local authorities in the UK were observed from one year to another with lower levels of pollution noted in the last 5 years of the observation window (2015–2019) in comparison to previous years (2009–2014), with an exception for the year 2016 (Fig. 4).

A high correlation (Pearson’s coefficient ≥ 0.7) was also observed between NO_2_, PM10, and PM2.5 pollutants at the local authority level (Table 3).

**Table 3:** Exposure description and correlation matrix of air pollutants at the local authority level (*N* = 391 local authorities)

	Pearson’s correlation coefficient							
	NO_2_ (µg/m^3^)	SO_2_ (µg/m^3^)	PM10 (µg/m^3^)	PM2.5 (µg/m^3^)	Mean	SD	Median	Interquartile range
NO_2_ (µg/m^3^)	1.00				12.99	7.08	11.71	8.18
SO_2_ (µg/m^3^)	0.50	1.00			1.57	0.93	1.37	0.90
PM10 (µg/m^3^)	**0.77**	0.38	1.00		14.06	3.19	14.43	4.26
PM2.5 (µg/m^3^)	**0.81**	0.42	**0.97**	1.00	9.42	2.35	9.62	3.23

Strong correlations with correlation coefficient ≥ 0.70 are highlighted in bold

### Description of individuals’ self-reported health

The mean of self-reported general health (1 to 5: excellent to poor) was 2.65 (SD = 0.36) indicating that most of the individuals report good health. Specifically, excellent/very good/good health was prevalent in 79% of the responses, while 21% of responses indicated fair/poor health. High homogeneity in the self-reported health responses was noted within the individual clusters over time (ICC = 0.65), while low homogeneity was observed within the household clusters (ICC = 0.24) (Table 4).

**Table 4:** Intraclass correlation coefficient for within individual and household clusters

		**General Health scale (1 to 5: excellent to poor)**
**Individual ID**	ICC [95%CI]	***0.65 [0.64, 0.65]***
	N of observations	404,264
	N of individuals	67,982
	Mean^a^ (SD)	2.65 (0.36)
**Household ID**	ICC [95%CI]	0.24 [0.24, 0.24]
	N of observations	404,264
	N of households	233,212

Strong ICCs > 0.3 are highlighted in italic-bold; Mean^a^ is based on predictions from mixed-effects linear models which are adjusted for age in fixed effects and for the individual ID in random intercept

### The spatial–temporal effect of air pollution on individuals’ health

#### The spatial–temporal effect of air pollution on individuals’ health at the LSOAs level

Results showed that poorer self-reported health (1–5 Likert scale: excellent to poor health) is associated with increased concentrations of air pollution linked at the LSOAs level. Individuals were 10% (95%CI = 7%-14%), 36% (95%CI = 26%-47%), 15% (95%CI = 8%-23%), and 25% (95%CI = 15%-36%) more likely to increase the rating of their general health by one point, moving from excellent to poor health, for every 10 µg/m^3^ increase in NO_2_, SO_2_, PM10, and PM2.5 pollutants, respectively (Table 5). Performing sensitivity analysis using four-level mixed-effects models also revealed similar results of higher odds of fair/poor health with increasing concentrations of all the four pollutants (Additional file 1: Supplementary Table 2). In bi-pollutant models adjusting each of NO_2_, PM10 and PM2.5 models for SO_2_ pollutant, similar results were observed of poorer self-reported health with increasing concentrations of NO_2_, PM10 and PM2.5 pollutants (Table 6).

**Table 5:** The association of self-reported general health with each of NO_2_, SO_2_, PM10, and PM2.5 air pollutants in separate models at the LSOAs level (*N* = 404,264 observations from 67,982 individuals)

	Model 1	Model 2	Model 3
	OR [95%CI]	OR [95%CI]	OR [95%CI]
**Overall pollution effect**			
NO_2_ (µg/m^3^)	1.20 [1.16, 1.23]**	1.11 [1.08, 1.14]**	1.10 [1.07, 1.14]**
SO_2_ (µg/m^3^)	1.46 [1.35, 1.58]**	1.37 [1.27, 1.48]**	1.36 [1.26, 1.47]**
PM10 (µg/m^3^)	1.28 [1.20, 1.37]**	1.19 [1.11, 1.26]**	1.15 [1.08, 1.23]**
PM2.5 (µg/m^3^)	1.42 [1.31, 1.55]**	1.29 [1.19, 1.40]**	1.25 [1.15, 1.36]**
**Between pollution effect**			
NO_2_ (µg/m^3^)	1.22 [1.18, 1.26]**	1.11 [1.07, 1.14]**	1.09 [1.05, 1.14]**
SO_2_ (µg/m^3^)	16.45 [12.61, 21.44]**	5.88 [4.65, 7.42]**	5.69 [4.50, 7.19]**
PM10 (µg/m^3^)	1.29 [1.19, 1.40]**	1.17 [1.08, 1.27]**	1.11 [1.02, 1.21]*
PM2.5 (µg/m^3^)	1.50 [1.34, 1.69]**	1.31 [1.18, 1.47]**	1.23 [1.09, 1.38]**
**Within pollution effect**			
NO_2_ (µg/m^3^)	1.04 [0.97, 1.12]	1.02 [0.95, 1.10]	1.02 [0.95, 1.10]
SO_2_ (µg/m^3^)	0.97 [0.85, 1.10]	0.99 [0.88, 1.12]	0.99 [0.88, 1.13]
PM10 (µg/m^3^)	1.12 [0.98, 1.27]	1.06 [0.93, 1.20]	1.05 [0.93, 1.20]
PM2.5 (µg/m^3^)	1.004 [0.86, 1.17]	0.93 [0.78, 1.08]	0.92 [0.79, 1.08]

ORs and 95%CIs are expressed in terms of 10 µg/m^3^ increase in the air pollutants; Model 1 is adjusted for age, gender, and year dummies (2009–2019); Model 2 is adjusted for age, gender, ethnicity, country of birth, marital status, education, occupation, housing tenure, perceived financial situation, smoking status, and year dummies (2009–2019); Model 3 is additionally adjusted for the LSOAs population density

^**^P-value < 0.01; *P-value < 0.05

**Table 6:** The association of self-reported general health with each of NO_2_, SO_2_, PM10, and PM2.5 air pollutants in bi-pollutant models adjusted for SO_2_ pollutant at the LSOAs level (*N* = 404,264 observations from 67,982 individuals)

		**Overall pollution effect**
		OR [95%CI]
**NO**_**2**_**—SO**_**2**_** Model**	NO_2_ (µg/m^3^)	1.07 [1.04, 1.11]**
	SO_2_ (µg/m^3^)	1.30 [1.20, 1.41]**
**PM10—SO**_**2**_** Model**	PM10 (µg/m^3^)	1.10 [1.03, 1.17]**
	SO_2_ (µg/m^3^)	1.33 [1.23, 1.45]**
**PM2.5—SO**_**2**_** Model**	PM2.5 (µg/m^3^)	1.18 [1.08, 1.29]**
	SO_2_ (µg/m^3^)	1.33 [1.22, 1.44]**

ORs and 95%CIs are expressed in terms of 10 µg/m^3^ increase in the air pollutants

Models are additionally adjusted for age, gender, ethnicity, country of birth, marital status, education, occupation, housing tenure, perceived financial situation, smoking status, year dummies (2009–2019), and LSOAs population density

^**^P-value < 0.01; *P-value < 0.05

Decomposing the overall effect of air pollution on health into *between* (spatial: across LSOAs) and *within* (temporal: across years within each LSOA) effects, showed significant positive associations with poorer health for the *between* effect for NO_2_ (OR = 1.09, 95%CI = 1.05–1.14), SO_2_ (OR = 5.69, 95%CI = 4.50–7.19), PM10 (OR = 1.11, 95%CI = 1.02–1.21), and PM2.5 (OR = 1.23, 95%CI = 1.09–1.38) pollutants. No significant *within* effects were observed for these four pollutants, although the sign of the odds ratios is largely as expected (Table 5). Similar results for the *overall* and for the *between-within* effects of the four pollutants on individuals’ health were shown in a sensitivity analysis for wave 1 recruited individuals (Additional file 1: Supplementary Table 3).

#### The spatial–temporal effect of air pollution on individuals’ health at the local authority level

At the local authority level, individuals were 8% (95%CI = 5%-12%), 28% (95%CI = 13%-45%), 10% (95%CI = 1%-19%), and 19% (95%CI = 7%-31%) more likely to increase the rating of their general health by one point, moving from excellent to poor health, for every 10 µg/m^3^ increase in NO_2_, SO_2_, PM10, and PM2.5 pollutants, respectively (Table 7). This shows that the higher the pollution levels in a local authority are the poorer the health of individuals living there is. Similar results were noted in bi-pollutant models adjusting each of the NO_2_, PM10 and PM2.5 models for the SO_2_ pollutant. An exception was PM10 which does not show a significant association with self-reported general health after adjusting for the SO_2_ pollutant (Table 8). This could be explained by the moderate correlation between PM10 and SO_2_ (Pearson’s coefficient = 0.38), when linked at the local authority level. This was not the case when air pollution was linked at the LSOAs level, where the correlation between PM10 and SO_2_ (Pearson’s coefficient = 0.28) was lower.

**Table 7:** The association of self-reported general health with each of NO_2_, SO_2_, PM10, and PM2.5 air pollutants in separate models at the local authority level (*N* = 404,264 observations from 67,982 individuals)

	Model 1	Model 2
	OR [95%CI]	OR [95%CI]
**Overall pollution effect**		
NO_2_ (µg/m^3^)	1.13 [1.09, 1.18]**	1.08 [1.05, 1.12]**
SO_2_ (µg/m^3^)	1.28 [1.14, 1.43]**	1.28 [1.13, 1.45]**
PM10 (µg/m^3^)	1.14 [1.05, 1.23]**	1.10 [1.01, 1.19]*
PM2.5 (µg/m^3^)	1.25 [1.13, 1.38]**	1.19 [1.07, 1.31]**
**Between pollution effect**		
NO_2_ (µg/m^3^)	1.12 [1.04, 1.20]**	1.05 [0.99, 1.10]
SO_2_ (µg/m^3^)	15.05 [7.77, 29.15]**	6.31 [3.46, 11.5]**
PM10 (µg/m^3^)	1.04 [0.87, 1.25]	1.03 [0.90, 1.18]
PM2.5 (µg/m^3^)	1.09 [0.85, 1.41]	1.06 [0.88, 1.29]
**Within pollution effect**		
NO_2_ (µg/m^3^)	1.01 [0.92, 1.12]	1.01 [0.91, 1.12]
SO_2_ (µg/m^3^)	1.05 [0.86, 1.28]	1.04 [0.83, 1.30]
PM10 (µg/m^3^)	1.03 [0.90, 1.19]	1.02 [0.88, 1.19]
PM2.5 (µg/m^3^)	0.87 [0.73, 1.02]	0.86 [0.72, 1.03]

ORs and 95%CIs are expressed in terms of 10 µg/m^3^ increase in the air pollutants; Model 1 is adjusted for age, gender, and year dummies (2009–2019); Model 2 is adjusted for age, gender, ethnicity, country of birth, marital status, education, occupation, housing tenure, perceived financial situation, smoking status, and year dummies (2009–2019)

^**^P-value < 0.01; *P-value < 0.05

**Table 8:** The association of self-reported general health with each of NO_2_, SO_2_, PM10, and PM2.5 air pollutants in bi-pollutant models adjusted for SO_2_ pollutant at the local authority level (*N* = 404,264 observations from 67,982 individuals)

		**Overall pollution effect**
		OR [95%CI]
**NO**_**2**_**—SO**_**2**_** Model**	NO_2_ (µg/m^3^)	1.07 [1.02, 1.11]**
	SO_2_ (µg/m^3^)	1.19 [1.03, 1.38]*
**PM10—SO**_**2**_** Model**	PM10 (µg/m^3^)	1.06 [0.98, 1.16]
	SO_2_ (µg/m^3^)	1.25 [1.10, 1.43]**
**PM2.5—SO**_**2**_** Model**	PM2.5 (µg/m^3^)	1.14 [1.02, 1.27]*
	SO_2_ (µg/m^3^)	1.24 [1.08, 1.42]**

ORs and 95%CIs are expressed in terms of 10 µg/m^3^ increase in the air pollutants; Models are additionally adjusted for age, gender, ethnicity, country of birth, marital status, education, occupation, housing tenure, perceived financial situation, smoking status, and year dummies (2009–2019)

^**^P-value < 0.01; *P-value < 0.05

Analysing the *between*-*within* effects revealed significant positive associations with poorer health for the *between* effect only for the SO_2_ (ORs = 6.31, 95%CI = 3.46–11.5) pollutant, while no significant *within* effect was noted for this pollutant. Contrary to the LSOAs, both *between* and *within* effects were not present for NO_2_, PM10 and PM2.5 pollutants at the local authority level (Table 7).

Sensitivity analysis for wave 1 recruited individuals showed similar results for the *overall* and for the *between-within* effects of the SO_2_ pollutant (at the local authority level) on individuals’ health. However, for wave 1 recruited individuals, PM10 and PM2.5 pollutants do not show an association with poor self-reported health and NO_2_ shows a significant *between* effect on self-reported health (Additional file 1: Supplementary Table 4).

### The association of air pollution with individuals’ health by ethnicity and country of birth

#### The association of air pollution with individuals’ health by ethnicity and country of birth at the LSOAs level

Examining the association between ethnicity and individuals’ health revealed poorer self-reported health among Indian (OR = 1.35, 95%CI = 1.21–1.50), Pakistani/Bangladeshi (OR = 1.82, 95%CI = 1.65–2.02), and mixed ethnicities (OR = 1.19, 95%CI = 1.04–1.36) in comparison to the British-white. On the contrary, other-white (OR = 0.86, 95%CI = 0.79–0.94) and Black/African/Caribbean (OR = 0.66, 95%CI = 0.59–0.73) showed better self-reported health than the British-white. Non-UK-born individuals also reported better health in comparison to UK-born individuals (OR = 0.85, 95%CI = 0.80–0.91), which is in line with the *“Healthy migrant effect”* theory (Additional file 1: Supplementary Table 1).

**Fig. 5:**
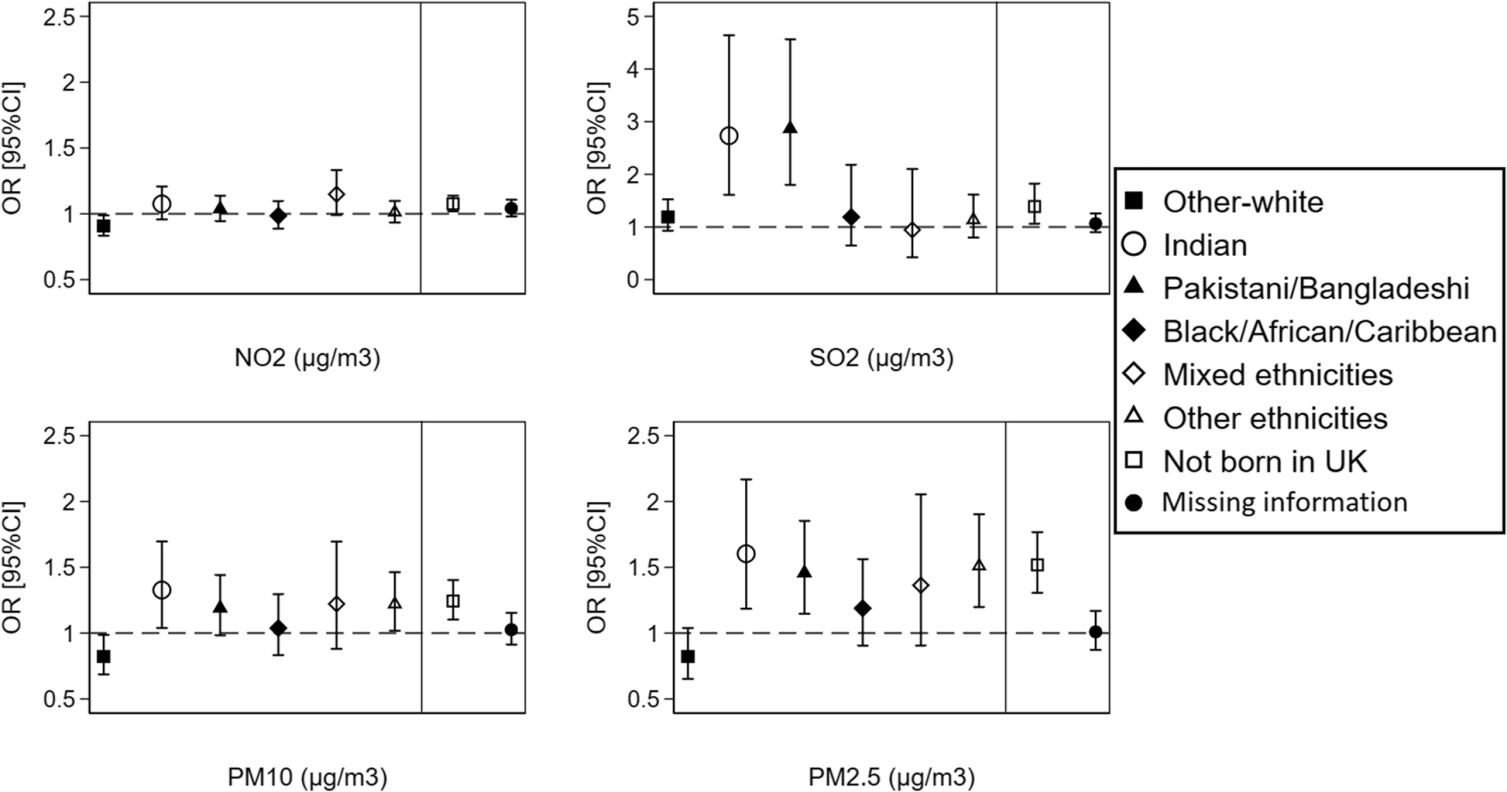
The overall effect of air pollution on individuals’ self-reported health by ethnicity and country of birth at the LSOAs level (*N* = 404,264 observations from 67,982 individuals). ORs and 95%CIs are expressed in terms of 10 µg/m^3^ increase in the air pollutants. The dashed line is placed at OR = 1 as a cut-off for statistically insignificant results; The solid line separates between the air pollution-ethnicity interaction models and the air pollution-country of birth interaction models; Air pollution-ethnicity interaction models where the reference category is “British-white” are adjusted for country of birth, age, gender, marital status, education, occupation, housing tenure, subjective financial situation, smoking status, year dummies (2009 to 2019), and LSOAs population density; Air pollution-country of birth interaction models where the reference category is “born in UK” are adjusted for ethnicity, age, gender, marital status, education, occupation, housing tenure, subjective financial situation, smoking status, year dummies (2009 to 2019), and LSOAs population density

Analysis of the association between air pollution and individuals’ health by ethnicity and country of birth at the LSOAs level showed a stronger effect of air pollution on poor self-reported health among ethnic minorities. Specifically, individuals from an Indian and Pakistani/Bangladeshi origins reported poorer health with every 10 µg/m^3^ increase in SO_2_, PM10, and PM2.5 pollutants compared to British-white. Non-UK-born individuals were also more likely to report poorer health than UK-born individuals with increasing concentrations of all four pollutants (Fig. 5). Similar results were observed in four-level mixed-effects models in which higher odds of fair/poor health were shown among people from Indian and Pakistani/Bangladeshi origins compared to British-white with increasing concentrations of SO_2_, PM10, and PM2.5 pollutants. Higher odds of fair/poor health were also observed among non-UK-born individuals compared to UK-born individuals with increasing concentrations of the four pollutants. However, Black/African/Caribbean ethnicities showed higher odds of fair/poor health compared to British-white while other-white showed lower odds of fair/poor health with increasing concentrations of NO_2_, PM10, and PM2.5 pollutants (Additional file 1: Supplementary Fig. 1). These associations were not observed in the three-level mixed-effects models, which included only a random intercept for LSOAs, but not for local authorities.

Sensitivity analysis for only wave 1 recruited individuals revealed similar associations, except for the association between PM10 pollutant and self-reported health, whereby no differences were shown among ethnic minorities and non-UK-born individuals compared to British-white and UK-born individuals (Additional file 1: Supplementary Fig. 2).

**Fig. 6:**
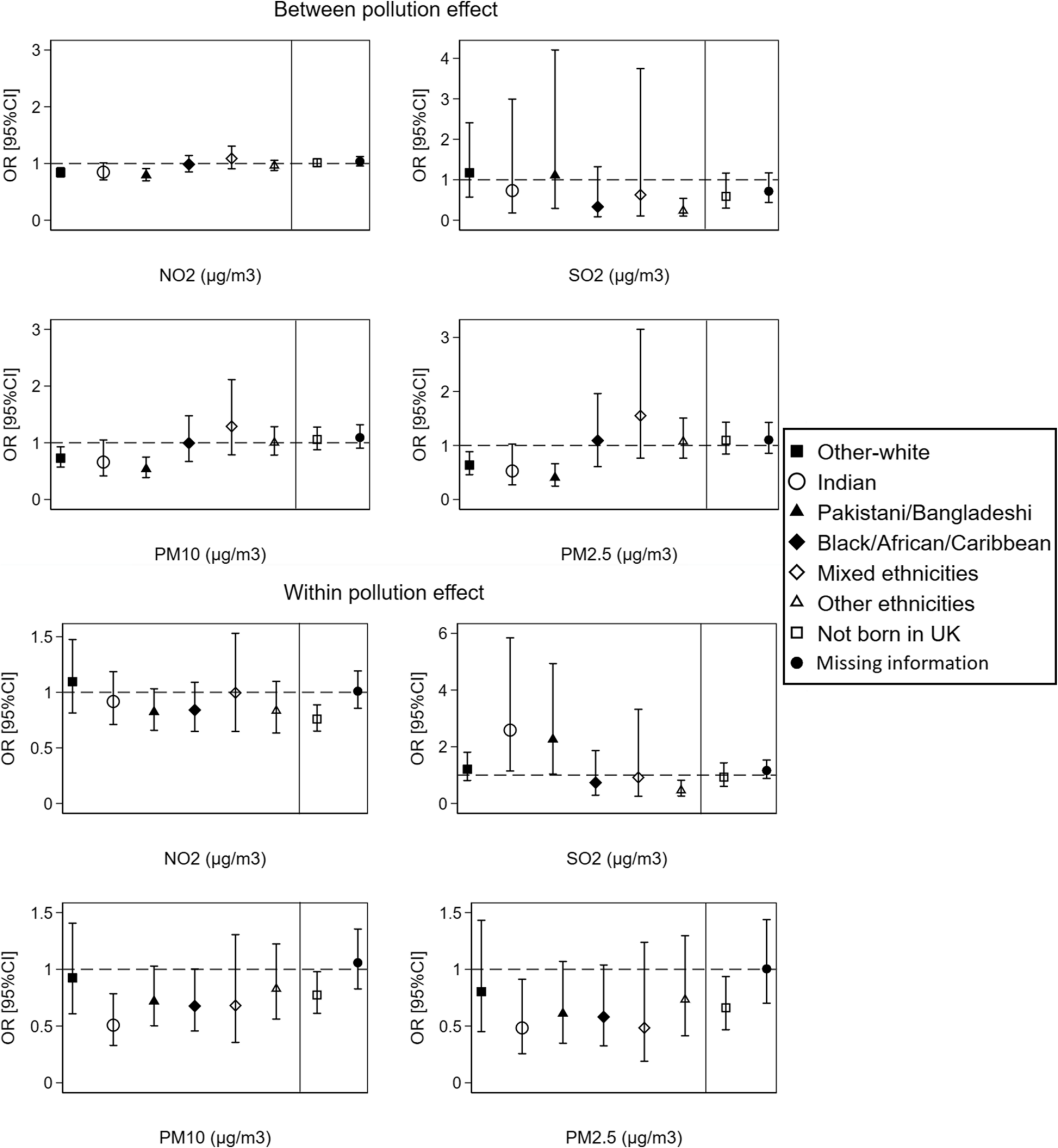
The *between-within* (spatial–temporal) effect of air pollution on individuals’ self-reported health by ethnicity and country of birth at the LSOAs level (*N* = 404,264 observations from 67,982 individuals). ORs and 95%CIs are expressed in terms of 10 µg/m^3^ increase in the air pollutants. The dashed line is placed at OR = 1 as a cut-off for statistically insignificant results; The solid line separates between the air pollution-ethnicity interaction models and the air pollution-country of birth interaction models; Air pollution-ethnicity interaction models where the reference category is “British-white” are adjusted for country of birth, age, gender, marital status, education, occupation, housing tenure, subjective financial situation, smoking status, year dummies (2009 to 2019), and LSOAs population density; Air pollution-country of birth interaction models where the reference category is “born in UK” are adjusted for ethnicity, age, gender, marital status, education, occupation, housing tenure, subjective financial situation, smoking status, year dummies (2009 to 2019), and LSOAs population density

Analysing the *between-within* (spatial–temporal) effects of air pollution on health by ethnicity and country of birth at the LSOAs level showed less consistent results than the overall effect of air pollution. Better health was reported with increasing LSOAs-11 years average concentrations of NO_2,_ PM10 and PM2.5 pollutants (*between* effect) by people from Pakistani/Bangladeshi origins compared to British-white. Indians and non-UK-born individuals also showed better health with more temporal variation (*within* effect) in PM10 and PM2.5 pollutants. In contrast, Indians and Pakistani/Bangladeshi ethnicities reported poorer health with more temporal variation in SO_2_ pollutant compared to British-white (Fig. 6). The *Between-within* analysis for individuals recruited at wave 1 of the Understanding Society study revealed similar results (Additional file 1: Supplementary Fig. 3).

#### The association of air pollution with individuals’ health by ethnicity and country of birth at the local authority level

**Fig. 7:**
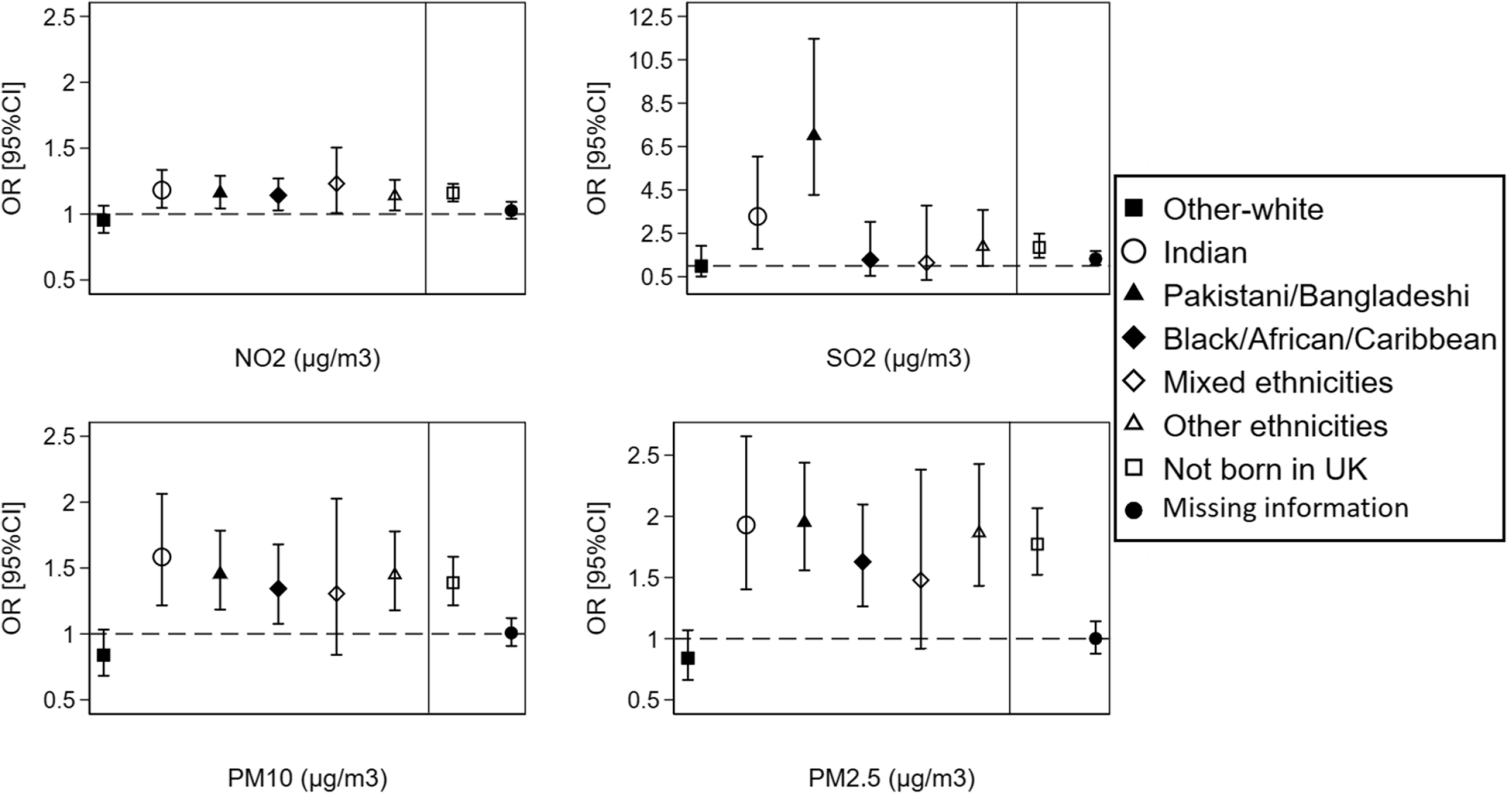
The overall effect of air pollution on individuals’ self-reported health by ethnicity and country of birth at the local authority level (*N* = 404,264 observations from 67,982 individuals). ORs and 95%CIs are expressed in terms of 10 µg/m^3^ increase in the air pollutants. The dashed line is placed at OR = 1 as a cut-off for statistically insignificant results; The solid line separates between the air pollution-ethnicity interaction models and the air pollution-country of birth interaction models; Air pollution-ethnicity interaction models where the reference category is “British-white” are adjusted for country of birth, age, gender, marital status, education, occupation, housing tenure, subjective financial situation, smoking status, and year dummies (2009 to 2019); Air pollution-country of birth interaction models where the reference category is “born in UK” are adjusted for ethnicity, age, gender, marital status, education, occupation, housing tenure, subjective financial situation, smoking status, and year dummies (2009 to 2019)

Analysis of air pollution and health by ethnicity and country of birth at the local authority level also revealed a stronger effect of air pollution on poor self-reported health among ethnic minorities; yet with some noted differences than the analysis performed at the LSOAs level. At the local authority level, individuals from an Indian, Pakistani/Bangladeshi, Black/African/Caribbean, and other ethnicities origin reported poorer health than the British-white with every 10 µg/m^3^ increase in NO_2_, PM10, and PM2.5 pollution. People from Indian and Pakistani/Bangladeshi origins also showed poorer health with increasing concentrations of SO_2_ pollutant (Fig. 7). Similar to LSOAs, non-UK-born individuals were more likely to report poorer health than UK-born individuals with increasing concentrations of all four pollutants linked at the local authority level (Fig. 7).

Similar association patterns were observed in the analysis for only wave 1 recruited individuals compared to the total sample analysis. However, the magnitude of the associations was reduced, and no significant differences are noted anymore between the ethnic minority groups and British-white for the association between NO_2_ and PM10 pollutants and self-reported health (Additional file 1: Supplementary Fig. 4). The most probable explanation for the reduced magnitude of associations is that by considering only wave 1 recruited individuals, we are missing the second ethnic minority boost sample, which was introduced at wave 6 of the data collection.

**Fig. 8:**
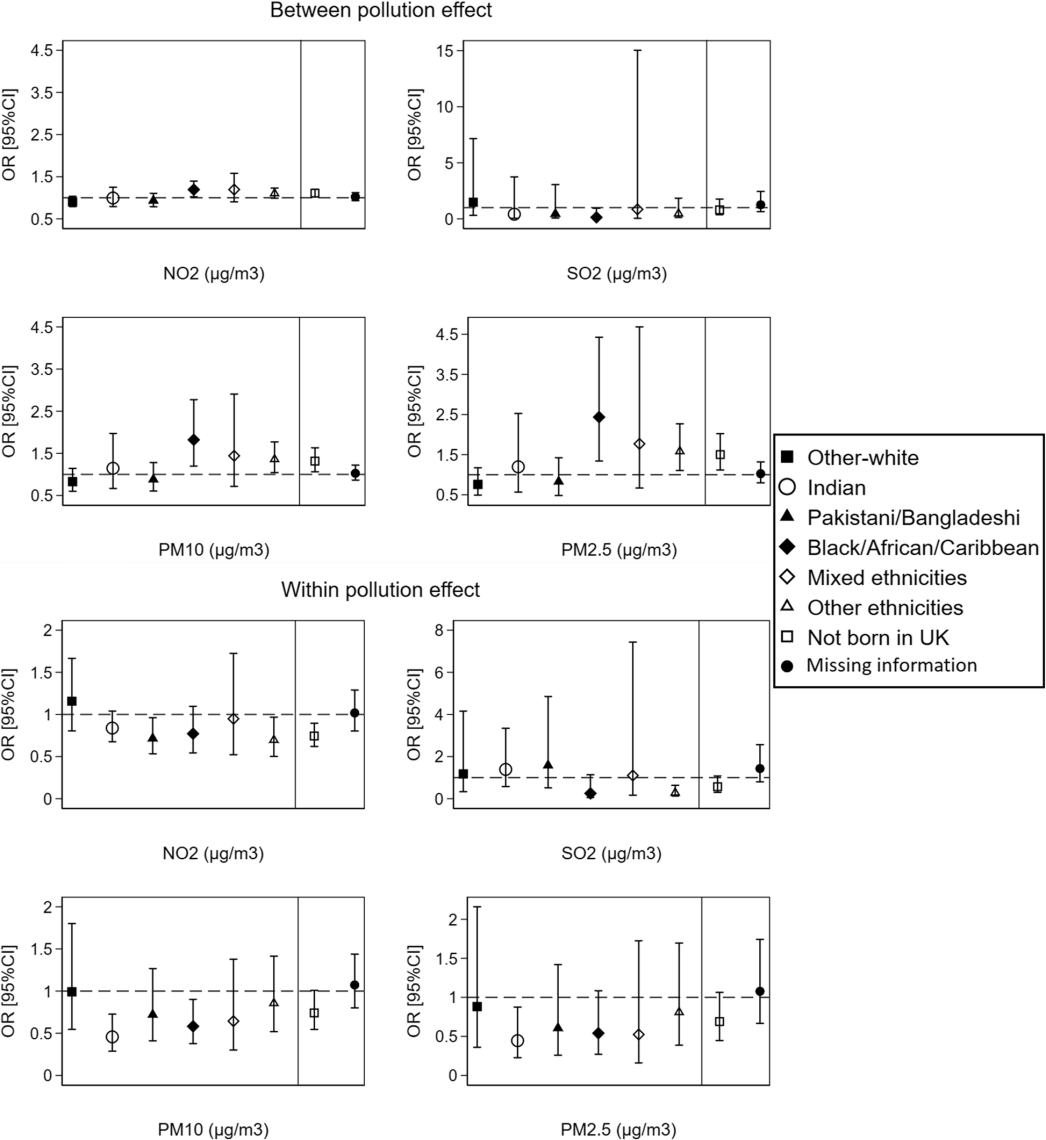
The *between-within* (spatial–temporal) effect of air pollution on individuals’ self-reported health by ethnicity and country of birth at the local authority level (*N* = 404,264 observations from 67,982 individuals). ORs and 95%CIs are expressed in terms of 10 µg/m^3^ increase in the air pollutants. The dashed line is placed at OR = 1 as a cut-off for statistically insignificant results; The solid line separates between the air pollution-ethnicity interaction models and the air pollution-country of birth interaction models; Air pollution-ethnicity interaction models where the reference category is “British-white” are adjusted for country of birth, age, gender, marital status, education, occupation, housing tenure, subjective financial situation, smoking status, and year dummies (2009 to 2019); Air pollution-country of birth interaction models where the reference category is “born in UK” are adjusted for ethnicity, age, gender, marital status, education, occupation, housing tenure, subjective financial situation, smoking status, and year dummies (2009 to 2019)

For the *between-within* (spatial–temporal) effects of air pollution at the local authority level, significant associations with poorer health were noted for the *between* effect of NO_2_, PM10, and PM2.5 pollution among the Black/African/Caribbean group in comparison to the British-white and among those not born in the UK. In contrast, Indians showed better health with more temporal variation (*within* effect) in PM10 and PM2.5 pollutants compared to British-white (Fig. 8). However, the above *Between-within* effects were not observed in the analysis for only wave 1 recruited individuals, except for Indians who still show better health with more temporal variation in PM10 pollutant (Additional file 1: Supplementary Fig. 5).

## Discussion

This study shows that there is an association between increased exposure to four air pollutants NO_2_, SO_2_, PM10, and PM2.5 (linked at coarse local authorities and detailed LSOAs geographical scales) and self-reported health in the UK for individuals followed between 2009 and 2019. These findings are corroborated by relevant literature whereby exposure to air pollution was associated with many respiratory (eg. asthma, bronchiolitis), cardiovascular (eg. chronic obstructive pulmonary disease, emphysema, myocardial infarction), cerebrovascular (eg. stroke), and cancer (eg. lung cancer) diseases [2, 4]. This in turn contributes to increased rates of mortality [54–57], hospital admissions [3, 15, 58, 59], and poor self-reported health [8, 10, 60].

Although the association between air pollution and poor health is well-established in the literature, this study was novel in going a further step in an attempt to show the *between-within* effects of air pollution on health. Additionally, the analysis was carried out at two geographical scales, the coarse local authorities and more detailed LSOAs, which forms another novelty of this study. The *between-within* analysis is widely used in the fields of economics, behavioural finance, and strategic management [61]. However, this type of analysis is rarely used in health research [62]; and no previous study has assessed the *between-within* effects of air pollution on health. Through the application of the between-within analysis in this study, we observed significant *between*, but not *within* effects on poor self-reported health for NO_2_ and SO_2_ pollutants, at both the LSOAs and local authority levels. However, for PM10 and PM2.5 pollutants, significant *between* but not *within* effects were observed only when linked at the LSOAs level, but not at the local authority level. Therefore, individuals residing in local authorities or LSOAs with higher average concentrations of NO_2_ and SO_2_ pollution across the 11 years of follow-up exhibited poorer self-reported health in comparison to individuals residing in local authorities or LSOAs with lower pollution concentrations. For particulate matter pollution, only residing in more polluted LSOAs resulted in poorer health. Hence, analysis at the local authority level attenuated the spatial (*between*) effect of PM10 and PM2.5 pollution on individuals’ health in comparison to the analysis at the LSOAs level. This implies stronger associations at the LSOAs finer geographical scale compared to the coarser local authority level. However, conducting analysis at the coarser local authorities’ level was necessary for guiding local authority-specific decision-making regarding air pollution and health.

In all cases, our study shows strong evidence for the spatial rather than temporal effects of air pollution on health, whether linked at the coarse local authority level or at the finer LSOAs level. This could be explained by the low variation of yearly air pollution concentrations across the 11 years of follow-up, particularly for SO_2_ pollutant as shown in Fig. 3 for LSOAs and Fig. 4 for local authorities. Hence, increasing the follow-up time to allow for more variation in air pollution might result in significant *within* effects. Additionally, air pollution exposure in this study was assessed on a yearly rather than monthly or daily basis, which also limits the variation in air pollution across time, resulting in weaker temporal associations.

Despite the statistically insignificant *within* results, the ORs for NO_2_, SO_2_, and PM10 indicated a positive association with poorer general health. This implies that the variation in air pollution over time within each local authority or LSOA can contribute to poorer health among individuals living in the respective local authority or LSOA. Hence, if the number of vehicles and/or industrial facilities increases over time in a respective local authority or LSOA, people may experience poorer health due to increased air pollution exposure.

The observed *between-within* effects can be also explained by the emission source of the pollutants and their chemical reactivity in the atmosphere. The major source of NO_2_ emissions is traffic exhaust [63], which varies across both local authorities/LSOAs (*between: spatial*) and time (*within: temporal*) depending on the number of vehicles and the movement of people. Yet, nitrogen oxides are highly reactive and seasonal pollutants [64], which makes it difficult to capture their temporal variation through yearly measurements. For instance, more NO_2_ will be liberated into the atmosphere during warm seasons due to the chemical reactions between nitrogen oxides and ozone [64]. Additionally, NO_2_ is converted to Nitric acid by several different reactions in the atmosphere [65]. That’s why only spatial (*between*) and not temporal (*within*) effects for NO_2_ pollutant were observed when taking the year as our time measuring unit.

On the other hand, industrial processes and power plants are the major sources of SO_2_ pollution [66], which is dominated by spatial (*between*) variation rather than temporal (*within*) variation as building a new factory requires much longer time than purchasing a motor vehicle. Particulate matter results from both traffic exhaust and industrial processes [67], and is considered a more stable pollutant that may stay suspended in the air for long periods of time [65]. Thus, an overall effect of particulate matter on health is expected rather than a spatial or a temporal derived effect. Yet, the stable nature of particulate matter allows this pollutant to show a spatial effect when using a high spatial resolution geographical scale such as LSOAs while this spatial effect will be attenuated when using a lower spatial resolution scale such as local authorities.

This study was also novel in analysing how the overall and the *between* and *within* effects of air pollution on self-reported health vary across six ethnic groups and by country of birth. Analysis revealed a stronger effect of air pollution on poor self-reported health among Pakistani/Bangladeshi, Indian, Black/African/Caribbean (only at the local authority level and in four-level nested models with a random intercept for repeated individual responses nested in LSOAs nested in local authorities), and other ethnic minorities compared to British-white; and among non-UK-born individuals compared to natives. These findings are corroborated by similar research from the United States of America whereby non-Hispanic white individuals were 10% more likely to report hypertension and non-Hispanic blacks were 2 times more likely to report asthma with increasing concentrations of PM2.5 pollution [7, 21].

In contrast, the *between-within* analysis did not show consistent associations between air pollution and health across the ethnic groups. Only individuals of Black/African/Caribbean origin and those not born in the UK reported poorer health with increasing concentrations of local authority-specific 11 years average NO_2_, PM10, and PM2.5 pollution (*between* effects). Whereas, better health was observed with more temporal variation in PM10 and PM2.5 pollutants (*within* effects) among Indians, and non-UK-born individuals.

The observed ethnic differences in health in the context of air pollution can be explained by two concepts derived from relevant literature on ethnic inequalities in health. The first concept relates to the socioeconomic and lifestyle behavioural differences among ethnic groups. Research has shown that ethnic minorities often live in more disadvantaged communities and have lower socioeconomic status, lower healthcare coverage, and higher job/income insecurity, which increases their risk of illness and leads to poor health [29, 31, 32]. People of Pakistani and Bangladeshi origins tend to report the poorest health in the UK, followed by people of Indian and Caribbean origins [30]. This was confirmed in our analysis whereby Pakistani/Bangladeshi, Indians, mixed, and other ethnicities individuals were more likely to report poor general health in comparison to British-white people (Additional file 1: Supplementary Table 1). However, our analysis accounted for major socioeconomic characteristics such as age, gender, marital status, education, occupation, housing tenure, and financial situation. Still, ethnic differences in the effect of air pollution on health persisted. Hence, those differences can be related to other socioeconomic and individual factors not captured in our analysis (e.g., genetics, racism and discrimination in healthcare access and patient services) or to contextual location-specific factors, which leads us to the second concept.

Contextual location-specific factors such as urbanisation, population density, neighbourhood, and housing conditions can help explain the observed ethnic differences in the effect of air pollution on health. Ethnic minorities and immigrants (foreign-born individuals) often reside in large cities and highly populated urbanised regions, near major roads and key transportation networks. This facilitates their movement and increases their chances of personal development, employment and business start-ups [68]. In addition, ethnic minorities often live in low-priced social housing offered by local authorities, which is often situated in more deprived ethnic concentration neighbourhoods or close to major roads and industrial areas [33]. In contrast, British-white and UK-born individuals are at a greater advantage in terms of job security, financial means, and inheritance tenure to move away from metropolitan areas and highly polluted industrial regions. Conducting a Chi2 tabulation in this study between ethnicity and housing tenure showed that around 24% of Pakistani/Bangladeshi, 45% of Black/African/Caribbean, and 29% of mixed ethnicities reside in houses rented from local authorities or housing associations compared to only 15% of British-white who live in these types of housing tenure (Additional file 1: Supplementary Table 5).

These location-specific factors would expose ethnic minorities and non-UK-born individuals to higher concentrations of air pollution related to traffic exhaust, industries, and burning of fossil fuels, which would manifest in greater health impacts compared to the rest of the population. In additional analysis through Chi2 tabulation, we show that a very high percentage of non-UK-born individuals (93.5%) and ethnic minorities including Pakistani/Bangladeshi (99.6%), Indian (98.4%), Black/African/Caribbean (98.9%), mixed (94.4%) and other ethnicities (84.0%) live in urban areas, whereas this percentage is much lower for British-white (71.5%) and UK-born (74.7%) individuals (Additional file 1: Supplementary Table 6). In a further analysis of individuals living in urban areas, we show that ethnic minorities and non-UK-born individuals live in more polluted local authorities especially for NO_2_ pollutant with an average exposure exceeding 20 µg/m^3^ for individuals from Pakistani/Bangladeshi, Indian, Black/African/Caribbean, and mixed ethnicity origins compared to an average exposure of 14 µg/m^3^ for the British-white group (Additional file 1: Supplementary Table 7). Furthermore, the *between-within* (*spatial–temporal*) analysis at the local authority level revealed stronger *between* effects for PM10 and PM2.5 pollution on poor self-reported health among Black/African/Caribbean and non-UK-born individuals. Thus, further confirming that residing in more polluted local authorities is a key explanation for the observed ethnic inequalities in health.

To sum up, living in deprived ethnic concentration areas which coincide with poor air quality is the most reasonable explanation for the observed ethnic inequalities in air pollution exposure and self-reported health. This is confirmed by Mitchell et al. (2015), whereby the most deprived areas in the UK still suffer from poor air quality despite the overall reduction in air pollution concentrations between 2001 and 2011 [39]. Thus, moving away from deprived areas would result in less exposure to air pollution among ethnic minorities, which would dilute the observed ethnic inequalities in self-reported health. One way to accomplish this is to reduce ethnic segregation and encourage ethnic diversity in the neighbourhoods. In this context, projections of the UK’s ethnic populations from 2001 to 2051 showed significant future changes with ethnic minorities increasing in size and share and shifting out of deprived local authorities into less deprived ones [69]. This was further confirmed by a recent analysis of the 2021 Census showing a growth of ethnic neighbourhood diversity across all the regions of England and Wales [70].

Despite the novelty of this study, it has some limitations. First, the assessment of individuals’ exposure to ambient air pollution was done using the local authority and LSOA of residence, which does not necessarily equate to the true personal exposure. In reality, an exposure scenario is more complex involving exposure indoors, at the workplace and through commuting patterns. Assessing the exposure at the local authorities might have helped in capturing some of these exposures such as exposures at the workplace or during commuting. However, this assumption stands only if the individual lives and works within the same local authority. Therefore, future studies are encouraged to integrate air pollution exposure at the residence and workplace (e.g., by using the residential and workplace postcodes) and to consider both ambient and indoor air pollution exposures.

Second, the smallest census area units were used to link the air pollution data to the “Understanding Society” individual-level data. Whilst these census areas offer a fine spatial resolution for the linkage of air pollution data, they have different classifications in the four nations of the UK based on a minimum population size quota and are called LSOAs in England and Wales, data zones in Scotland, and Super Output Areas in Northern Ireland. The potential size heterogeneity issue between the different census areas was addressed in our analysis by including a random intercept for those census areas referred jointly to as LSOAs. Additionally, we performed a set of models which are adjusted for the population density at the LSOAs level, and results remained unchanged. Not to mention that analysis was also done at the local authority level, a more harmonised geographical level compared to census areas, where similar results were shown. However, analysis at the local authority level and in four-level nested models which included a random intercept for both LSOAs and local authorities showed a stronger association between air pollution and health among the Black/African/Caribbean ethnicity, which was not the case for the analysis at the LSOAs level. This shows the importance of performing analysis at two geographical levels to disentangle the local and regional effects of air pollution on health, especially in the context of ethnicity.

Third, our study examined the association between air pollution and self-reported health rather than using more objective health measures such as mortality or hospital admissions. This could lead to social desirability or reporting bias whereby individuals overestimate or underestimate their general health. However, high correlations between self-reported health and objective health measures including mortality and hospital admissions were demonstrated by relevant literature, which increases the reliability of the self-reported health variable [11–13]. Furthermore, research from the UK has shown an association between poorer self-rated health and greater morbidity within each ethnic group; hence, providing evidence that the use of self-rated health to measure health status in different ethnic groups in the UK is valid [71].

Fourth, our study included all individuals recruited at different waves of the “Understanding society” data, that had at least two observations through the follow-up period (2009–2019). Therefore, some individuals were followed for the whole observation window of 11 years and started at wave 1 while others were recruited at later data collection waves and followed for a shorter period. Nevertheless, we performed sensitivity analysis only on individuals recruited in wave 1 to balance the cohort effect (Additional file 1: Supplementary Tables 3 and 4, and Additional file 1: Supplementary Figs. 2, 3, 4, and 5), and results remained unchanged, except for the analysis by ethnicity in which the magnitude of associations was reduced.

Fifth, the sample design of the “Understanding Society” survey involved ethnic minority boost samples at waves 1 and 6 of data collection to enable ethnicity-focused research. Thus, the survey included longitudinal weights that adjust for the overrepresentation of some ethnic groups. However, we could not adjust our analysis for the longitudinal weights as this requires that all individuals be followed until the last wave (wave 10) of the survey, which was not the case. Hence, our ethnicity analysis might not be generalizable to the whole UK population, but rather represent regions with dense ethnic minority concentrations.

Finally, our study included individuals followed over 11 years (2009–2019) of time. However, the air pollution variation across these 11 years was low, which did not allow for the detection of significant temporal (*within*) effects of air pollution on health. For future research, we recommend using other datasets with a longer follow-up time to allow for more variation in air pollution, which might result in significant temporal effects.

## Conclusion

Using a longitudinal panel design that involves linking individual-level to air pollution data at two geographical scales (coarse local authorities and detailed LSOAs), this study supports the presence of a spatial–temporal association between air pollution and individuals’ reported health in the UK. However, results showed stronger *between* (spatial) effects across local authorities/LSOAs rather than *within* (temporal) effects across time within each local authority/LSOA. Furthermore, this study demonstrates a stronger effect of air pollution on poor self-reported health among ethnic minorities and non-UK-born individuals, which is partly explained by location-specific differences. Our results are of importance for policymakers in the UK toward advancing legislations related to air pollution, health, time, and place with an emphasis on targeting the ethnic inequalities in air pollution exposure and health.

## Supplementary Information

**Additional file 1:** Sensitivity checks and additional analysis.

## Data Availability

We cannot make the data underlying our analysis publicly available due to ethical and legal restrictions. We are using the “Understanding Society: The UK Household Longitudinal Study” dataset which is an initiative funded by the Economic and Social Research Council and various Government Departments, with scientific leadership by the Institute for Social and Economic Research, University of Essex, and survey delivery by NatCen Social Research and Kantar Public. These data are protected by a copyright license and strictly distributed by the UK Data Service which is the largest digital repository for quantitative and qualitative social science and humanities research data in the UK. Therefore, data underlying our analysis can only be accessed through the UK Data Service for authorized researchers from the following URL: https://beta.ukdataservice.ac.uk/datacatalogue/series/series?id=2000053

## Abbreviations

NO_2_: Nitrogen dioxide
SO_2_: Sulphur dioxide
PM10: Particulate matter with a diameter ≤ 10 µm
PM2.5: Particulate matter with a diameter ≤ 2.5 µm
UK: United Kingdom
COMEAP: Committee on the Medical Effects of Air Pollution
LSOAs: Lower Super Output Areas
OR: Odd Ratio
CI: Confidence Interval
SD: Standard deviation
ICC: Intraclass correlation coefficient
