# Supplementary material for: The spatial–temporal effect of air pollution on individuals’ reported health and its variation by ethnic groups in the United Kingdom: a multilevel longitudinal analysis
Source: BMC Public Health. 2023 May 16;23:897. doi: 10.1186/s12889-023-15853-y (PMC10186783; doi:10.1186/s12889-023-15853-y)
Supplement: Supplementary file 1 — Additional file 1: Sensitivity checks and additional analysis. [file 12889_2023_15853_MOESM1_ESM.docx]

**Additional file 1: Sensitivity checks and additional analysis.**

Supplementary Table 1: The association of self-reported general health with the socio-demographic and lifestyle covariates (N=404,264 observations from 67,982 individuals)

|  |  | **OR** | **P-value** | **Lower 95%CI** | **Upper 95% CI** |
| --- | --- | --- | --- | --- | --- |
| **Ethnicity** | British-white | Ref | | | |
|  | Other-white | 0.86 | 0.001 | 0.79 | 0.94 |
|  | Indian | 1.35 | 0.000 | 1.21 | 1.50 |
|  | Pakistani/Bangladeshi | 1.82 | 0.000 | 1.65 | 2.02 |
|  | Black/African/Caribbean | 0.66 | 0.000 | 0.59 | 0.73 |
|  | Mixed ethnicities | 1.19 | 0.012 | 1.04 | 1.36 |
|  | Other ethnicities | 1.08 | 0.056 | 1.00 | 1.18 |
| **Country of birth** | Born in the UK | Ref | | | |
|  | Not born in the UK | 0.85 | 0.000 | 0.80 | 0.91 |
|  | Missing information | 0.72 | 0.000 | 0.68 | 0.77 |
| **Age** | 16-18 | Ref | | | |
|  | 19-23 | 1.11 | 0.000 | 1.06 | 1.17 |
|  | 24-28 | 1.32 | 0.000 | 1.23 | 1.41 |
|  | 29-33 | 1.68 | 0.000 | 1.55 | 1.81 |
|  | 34-38 | 2.14 | 0.000 | 1.98 | 2.30 |
|  | 39-43 | 2.89 | 0.000 | 2.68 | 3.13 |
|  | 44-48 | 4.00 | 0.000 | 3.70 | 4.32 |
|  | 49-53 | 5.74 | 0.000 | 5.30 | 6.21 |
|  | 54-58 | 7.47 | 0.000 | 6.88 | 8.10 |
|  | 59-63 | 8.83 | 0.000 | 8.11 | 9.60 |
|  | 64-68 | 8.94 | 0.000 | 8.19 | 9.76 |
|  | 69-73 | 10.57 | 0.000 | 9.65 | 11.58 |
|  | 74-78 | 15.19 | 0.000 | 13.78 | 16.74 |
|  | >78 | 23.71 | 0.000 | 21.35 | 26.34 |
| **Gender** | Male | Ref | | | |
|  | Female | 1.20 | 0.000 | 1.15 | 1.24 |
| **Education** | University degree | Ref | | | |
|  | High school degree | 1.52 | 0.000 | 1.45 | 1.58 |
|  | Lower educational levels | 1.90 | 0.000 | 1.63 | 2.21 |
|  | Other qualifications | 2.42 | 0.000 | 2.30 | 2.54 |
|  | Still a student | 0.89 | 0.000 | 0.84 | 0.94 |
| **Marital status** | Married | Ref | | | |
|  | Living as a couple | 1.13 | 0.000 | 1.08 | 1.18 |
|  | Widowed | 1.21 | 0.000 | 1.14 | 1.29 |
|  | Divorced/separated | 1.28 | 0.000 | 1.22 | 1.35 |
|  | Single never married | 1.15 | 0.000 | 1.10 | 1.21 |
|  | Missing information | 1.29 | 0.005 | 1.08 | 1.54 |
| **Occupation** | Managers/Professionals/employers | Ref | | | |
|  | Non manual workers | 1.18 | 0.000 | 1.13 | 1.22 |
|  | Manual workers | 1.10 | 0.000 | 1.05 | 1.14 |
|  | Not applicable: Student/ retired/Not working | 1.95 | 0.000 | 1.87 | 2.03 |
|  | Missing information | 1.14 | 0.002 | 1.05 | 1.25 |
| **Housing tenure** | Owned outright | Ref | | | |
|  | Owned with mortgage | 1.11 | 0.000 | 1.07 | 1.14 |
|  | Local authority rent | 2.64 | 0.000 | 2.50 | 2.79 |
|  | Housing association rent | 2.59 | 0.000 | 2.44 | 2.75 |
|  | Private rent | 1.64 | 0.000 | 1.56 | 1.73 |
|  | Other or missing information | 1.38 | 0.000 | 1.29 | 1.47 |
| **Subjective financial situation** | Living comfortably/doing alright | Ref | | | |
|  | Living difficultly | 1.63 | 0.000 | 1.59 | 1.66 |
|  | Missing information | 0.99 | 0.671 | 0.94 | 1.04 |
| **Smoking status** | Non smoker | Ref | | | |
|  | Smoker | 1.58 | 0.000 | 1.53 | 1.63 |
|  | Missing information | 1.00 | 0.963 | 0.96 | 1.04 |
| **Time dummies** | 2009 | Ref | | | |
|  | 2010 | 0.99 | 0.687 | 0.96 | 1.03 |
|  | 2011 | 0.99 | 0.458 | 0.95 | 1.02 |
|  | 2012 | 1.01 | 0.569 | 0.97 | 1.05 |
|  | 2013 | 1.02 | 0.234 | 0.99 | 1.06 |
|  | 2014 | 0.97 | 0.187 | 0.94 | 1.01 |
|  | 2015 | 1.08 | 0.000 | 1.04 | 1.12 |
|  | 2016 | 1.33 | 0.000 | 1.28 | 1.39 |
|  | 2017 | 1.51 | 0.000 | 1.45 | 1.57 |
|  | 2018 | 1.70 | 0.000 | 1.63 | 1.77 |
|  | 2019 | 1.77 | 0.000 | 1.68 | 1.86 |
| **LSOAs population density** | Continuous variable | 1.00 | 0.000 | 1.00 | 1.00 |

Supplementary Table 2: The association of self-reported general health measured as a binary variable (0=Excellent/very good/good health; 1=Fair/poor health) with each of NO_2_, SO_2_, PM10, and PM2.5 air pollutants in separate four-level mixed-effects logistic models with a random intercept for repeated individual observations nested in LSOAs, nested in local authorities (N=404,264 observations from 67,982 individuals)

|  | **Overall pollution effect** |
| --- | --- |
|  | OR [95%CI] |
| NO_2_ (µg/m^3^) | 1.11 [1.05, 1.17]** |
| SO_2_ (µg/m^3^) | 1.33 [1.17, 1.53]** |
| PM10 (µg/m^3^) | 1.18 [1.05, 1.33]** |
| PM2.5 (µg/m^3^) | 1.31 [1.12, 1.53]** |

**P-value <0.01; *P-value<0.05;

ORs and 95%CIs are expressed in terms of 10 µg/m^3^ increase in the air pollutants linked at the LSOAs level; Models are adjusted for age, gender, ethnicity, country of birth, marital status, education, occupation, housing tenure, perceived financial situation, smoking status, year dummies (2009-2019), and LSOAs population density.

Supplementary Figure 1: The association of self-reported general health measured as a binary variable (0=Excellent/very good/good health; 1=Fair/poor health) with each of NO_2_, SO_2_, PM10, and PM2.5 air pollutants by ethnicity and country of birth in separate four-level mixed-effects logistic models with a random intercept for repeated individual observations nested in LSOAs, nested in local authorities (N=404,264 observations from 67,982 individuals)
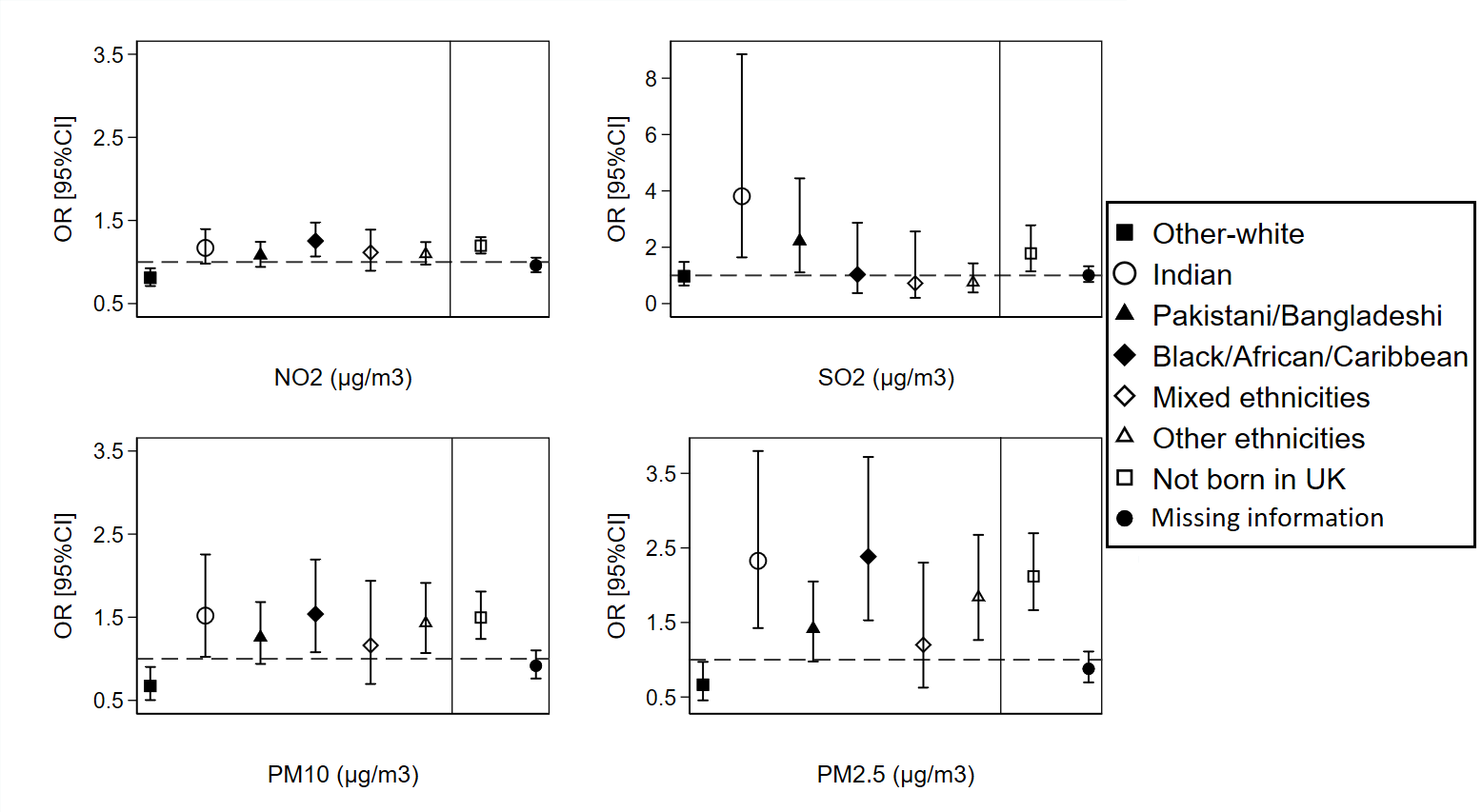
ORs and 95%CIs are expressed in terms of 10 µg/m^3^ increase in the air pollutants;

The dashed line is placed at OR=1 as a cut-off for statistically insignificant results; The solid line separates between the air pollution-ethnicity interaction models and the air pollution-country of birth interaction models; Air pollution-ethnicity interaction models where the reference category is “British-white” are adjusted for country of birth, age, gender, marital status, education, occupation, housing tenure, subjective financial situation, smoking status, year dummies (2009 to 2019), and LSOAs population density; Air pollution-country of birth interaction models where the reference category is “born in UK” are adjusted for ethnicity, age, gender, marital status, education, occupation, housing tenure, subjective financial situation, smoking status, year dummies (2009 to 2019), and LSOAs population density.

Supplementary Table 3: The association of self-reported general health with each of NO_2_, SO_2_, PM10, and PM2.5 air pollutants for individuals recruited in wave 1 of the Understanding Society study at the LSOAs level (N=261,271 observations from 39,798 individuals)

|  | Model 1 | Model 2 | Model 3 |
| --- | --- | --- | --- |
|  | OR [95%CI] | OR [95%CI] | OR [95%CI] |
| **Overall pollution effect** | | |  |
| NO_2_ (µg/m^3^) | 1.24 [1.20, 1.29]** | 1.12 [1.09, 1.16]** | 1.12 [1.08, 1.16]** |
| SO_2_ (µg/m^3^) | 1.48 [1.34, 1.63]** | 1.39 [1.26, 1.53]** | 1.38 [1.25, 1.52]** |
| PM10 (µg/m^3^) | 1.33 [1.23, 1.44]** | 1.18 [1.09, 1.27]** | 1.14 [1.05, 1.24]** |
| PM2.5 (µg/m^3^) | 1.52 [1.37, 1.68]** | 1.30 [1.17, 1.43]** | 1.25 [1.12, 1.39]** |
| **Between pollution effect** | | |  |
| NO_2_ (µg/m^3^) | 1.28 [1.23, 1.34]** | 1.13 [1.08, 1.17]** | 1.12 [1.06, 1.17]** |
| SO_2_ (µg/m^3^) | 26.80 [19.20, 37.42]** | 7.47 [5.57, 10.02]** | 7.23 [5.38, 9.71]** |
| PM10 (µg/m^3^) | 1.40 [1.26, 1.55]** | 1.19 [1.08, 1.31]** | 1.13 [1.01, 1.25]* |
| PM2.5 (µg/m^3^) | 1.69 [1.46, 1.95]** | 1.36 [1.18, 1.56]** | 1.26 [1.09, 1.47]** |
| **Within pollution effect** | | |  |
| NO_2_ (µg/m^3^) | 1.04 [0.95, 1.14] | 1.03 [0.94, 1.12] | 1.03 [0.94, 1.13] |
| SO_2_ (µg/m^3^) | 0.94 [0.81, 1.10] | 0.99 [0.86, 1.16] | 1.00 [0.86, 1.17] |
| PM10 (µg/m^3^) | 1.12 [0.96, 1.32] | 1.04 [0.89, 1.22] | 1.04 [0.89, 1.22] |
| PM2.5 (µg/m^3^) | 1.01 [0.83, 1.22] | 0.91 [0.75, 1.11] | 0.91 [0.75, 1.10] |

**P-value <0.01; *P-value<0.05;

ORs and 95%CIs are expressed in terms of 10 µg/m^3^ increase in the air pollutants;

Model 1: is adjusted for age, gender, and year dummies (2009-2019); Model 2 is adjusted for age, gender, ethnicity, country of birth, marital status, education, occupation, housing tenure, perceived financial situation, smoking status, and year dummies (2009-2019); Model 3 is additionally adjusted for the LSOAs population density.

Supplementary Table 4: The association of self-reported general health with each of NO_2_, SO_2_, PM10, and PM2.5 air pollutants for individuals recruited in wave 1 of the Understanding Society study at the local authority level (N=261,271 observations from 39,798 individuals)

|  | Model 1 | Model 2 |
| --- | --- | --- |
|  | OR [95%CI] | OR [95%CI] |
| **Overall pollution effect** | | |
| NO_2_ (µg/m^3^) | 1.15 [1.10, 1.21]** | 1.09 [1.04, 1.14]** |
| SO_2_ (µg/m^3^) | 1.15 [1.02, 1.31]* | 1.20 [1.03, 1.41]* |
| PM10 (µg/m^3^) | 1.09 [0.98, 1.20] | 1.05 [0.95, 1.15] |
| PM2.5 (µg/m^3^) | 1.19 [1.05, 1.36]** | 1.12 [0.99, 1.26] |
| **Between pollution effect** | | |
| NO_2_ (µg/m^3^) | 1.17 [1.07, 1.26]** | 1.07 [1.01, 1.14]* |
| SO_2_ (µg/m^3^) | 24.05 [11.26, 51.37]** | 8.16 [4.54, 12.6]** |
| PM10 (µg/m^3^) | 1.07 [0.86, 1.32] | 1.04 [0.89, 1.21] |
| PM2.5 (µg/m^3^) | 1.15 [0.85, 1.56] | 1.10 [0.88, 1.37] |
| **Within pollution effect** | | |
| NO_2_ (µg/m^3^) | 1.03 [0.92, 1.15] | 1.04 [0.93, 1.17] |
| SO_2_ (µg/m^3^) | 1.01 [0.76, 1.34] | 1.03 [0.82, 1.30] |
| PM10 (µg/m^3^) | 1.04 [0.85, 1.26] | 1.02 [0.83, 1.25] |
| PM2.5 (µg/m^3^) | 0.82 [0.66, 1.03] | 0.82 [0.65, 1.03] |

**P-value <0.01; *P-value<0.05;

ORs and 95%CIs are expressed in terms of 10 µg/m^3^ increase in the air pollutants; Model 1 is adjusted for age, gender, and year dummies (2009-2019); Model 2 is adjusted for age, gender, ethnicity, country of birth, marital status, education, occupation, housing tenure, perceived financial situation, smoking status, and year dummies (2009-2019).

Supplementary Figure 2: The overall effect of air pollution on individuals’ self-reported health by ethnicity and country of birth for individuals recruited in wave 1 of the Understanding Society study at the LSOAs level (N=261,271 observations from 39,798 individuals)


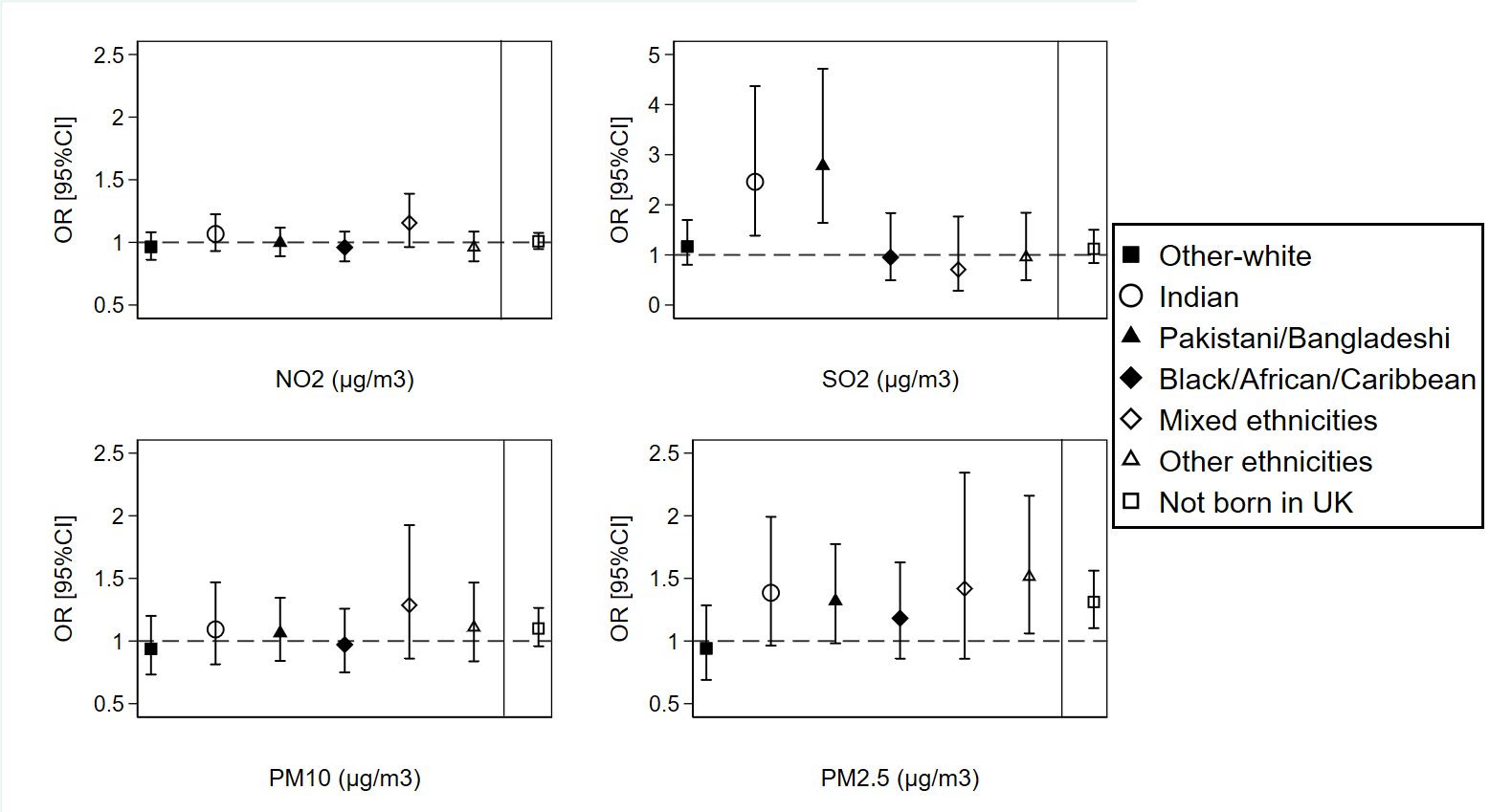


ORs and 95%CIs are expressed in terms of 10 µg/m^3^ increase in the air pollutants;

The dashed line is placed at OR=1 as a cut-off for statistically insignificant results; The solid line separates between the air pollution-ethnicity interaction models and the air pollution-country of birth interaction models; Air pollution-ethnicity interaction models where the reference category is “British-white” are adjusted for country of birth, age, gender, marital status, education, occupation, housing tenure, subjective financial situation, smoking status, year dummies (2009 to 2019), and LSOAs population density; Air pollution-country of birth interaction models where the reference category is “born in UK” are adjusted for ethnicity, age, gender, marital status, education, occupation, housing tenure, subjective financial situation, smoking status, year dummies (2009 to 2019), and LSOAs population density.

Supplementary Figure 3: The *between-within* (spatial-temporal) effect of air pollution on individuals’ self-reported health by ethnicity and country of birth for individuals recruited in wave 1 of the Understanding Society study at the LSOAs level (N=261,271 observations from 39,798 individuals)


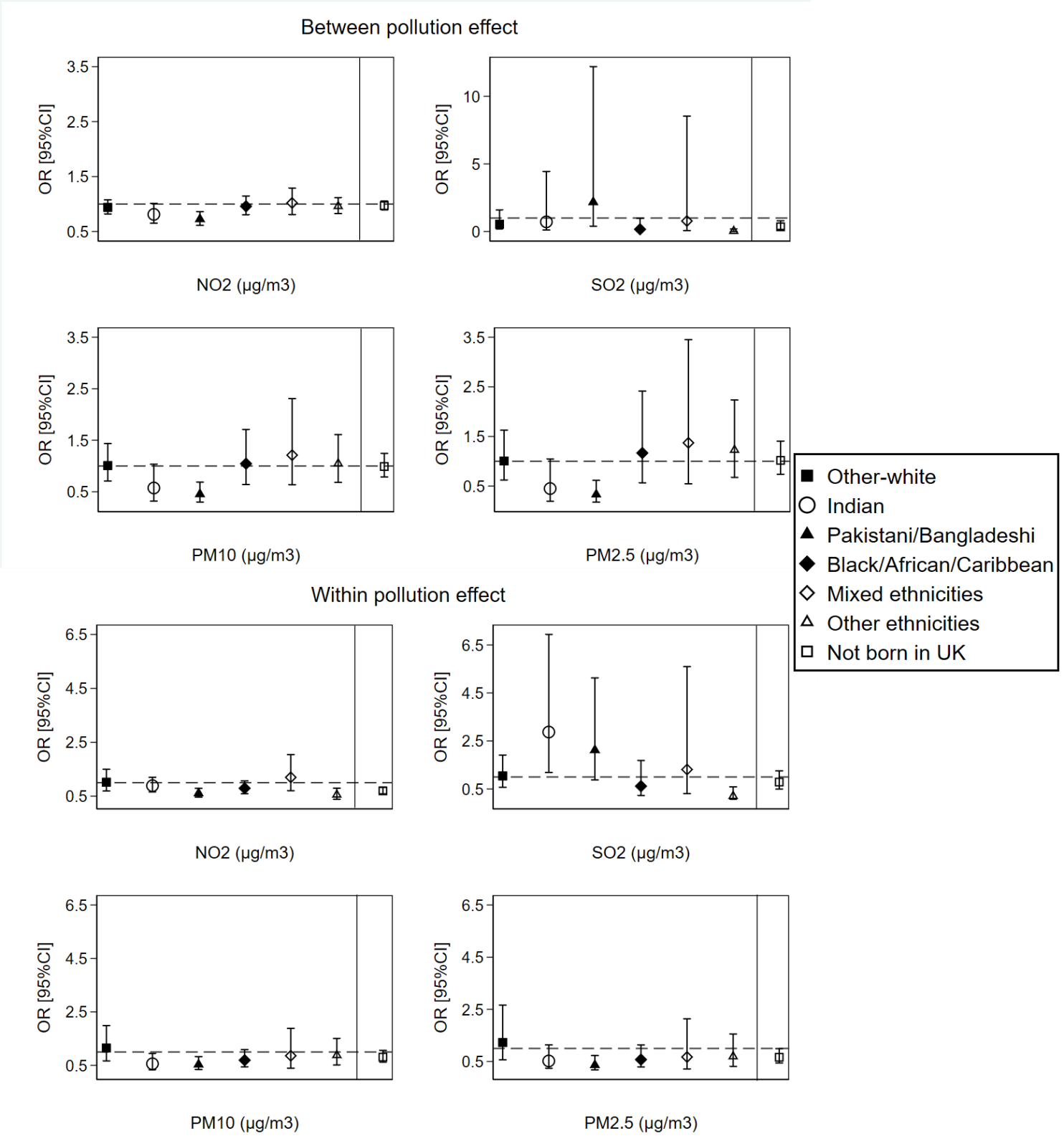


ORs and 95%CIs are expressed in terms of 10 µg/m^3^ increase in the air pollutants;

The dashed line is placed at OR=1 as a cut-off for statistically insignificant results; The solid line separates between the air pollution-ethnicity interaction models and the air pollution-country of birth interaction models; Air pollution-ethnicity interaction models where the reference category is “British-white” are adjusted for country of birth, age, gender, marital status, education, occupation, housing tenure, subjective financial situation, smoking status, year dummies (2009 to 2019), and LSOAs population density; Air pollution-country of birth interaction models where the reference category is “born in UK” are adjusted for ethnicity, age, gender, marital status, education, occupation, housing tenure, subjective financial situation, smoking status, year dummies (2009 to 2019), and LSOAs population density.

Supplementary Figure 4: The overall effect of air pollution on individuals’ self-reported health by ethnicity and country of birth for individuals recruited in wave 1 of the Understanding Society study at the local authority level (N=261,271 observations from 39,798 individuals)


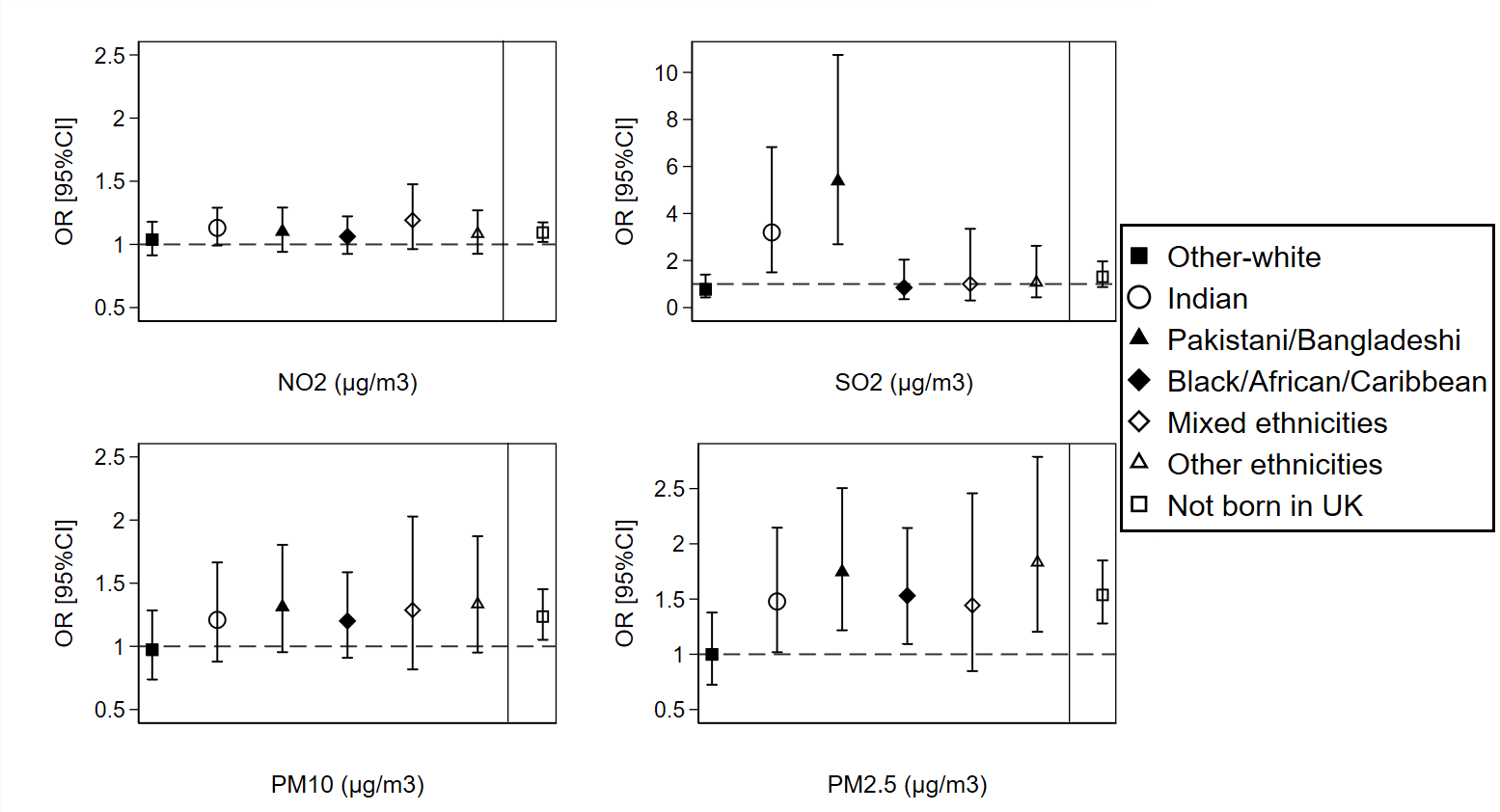


ORs and 95%CIs are expressed in terms of 10 µg/m^3^ increase in the air pollutants;

The dashed line is placed at OR=1 as a cut-off for statistically insignificant results; The solid line separates between the air pollution-ethnicity interaction models and the air pollution-country of birth interaction models; Air pollution-ethnicity interaction models where the reference category is “British-white” are adjusted for country of birth, age, gender, marital status, education, occupation, housing tenure, subjective financial situation, smoking status, and year dummies (2009 to 2019); Air pollution-country of birth interaction models where the reference category is “born in UK” are adjusted for ethnicity, age, gender, marital status, education, occupation, housing tenure, subjective financial situation, smoking status, and year dummies (2009 to 2019).

Supplementary Figure 5: The *between-within* (spatial-temporal) effect of air pollution on individuals’ self-reported health by ethnicity and country of birth for individuals recruited in wave 1 of the Understanding Society study at the local authority level (N=261,271 observations from 39,798 individuals)


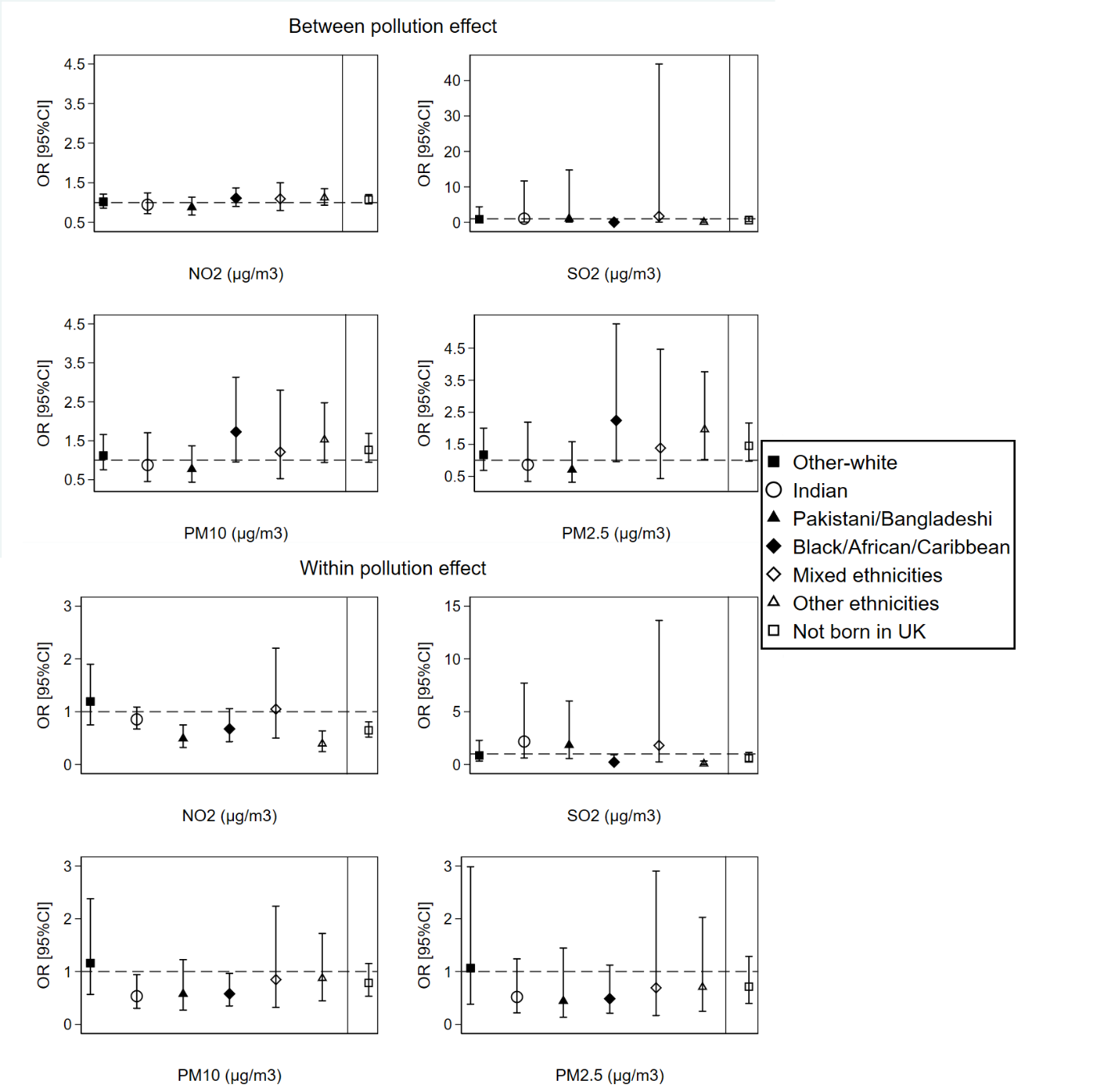


ORs and 95%CIs are expressed in terms of 10 µg/m^3^ increase in the air pollutants;

The dashed line is placed at OR=1 as a cut-off for statistically insignificant results; The solid line separates between the air pollution-ethnicity interaction models and the air pollution-country of birth interaction models; Air pollution-ethnicity interaction models where the reference category is “British-white” are adjusted for country of birth, age, gender, marital status, education, occupation, housing tenure, subjective financial situation, smoking status, and year dummies (2009 to 2019); Air pollution-country of birth interaction models where the reference category is “born in UK” are adjusted for ethnicity, age, gender, marital status, education, occupation, housing tenure, subjective financial situation, smoking status, and year dummies (2009 to 2019).

|  | | Owned outright | Owned with mortgage | Local authority rent | Housing association rent | Private rent | Other or missing information | Total |
| --- | --- | --- | --- | --- | --- | --- | --- | --- |
| Ethnicity (Chi2 test P-value=0.000) | | | | | | | | |
| British-white | Count | 111,428 | 118,302 | 26,343 | 18,901 | 30,978 | 3,640 | 309,592 |
|  | % | 35.99 | 38.21 | 8.51 | 6.11 | 10.01 | 1.18 | 100 |
| Other-white | Count | 5,170 | 7,134 | 1,929 | 936 | 4,248 | 333 | 19,750 |
|  | % | 26.18 | 36.12 | 9.77 | 4.74 | 21.51 | 1.69 | 100 |
| Indian | Count | 4,403 | 6,327 | 623 | 349 | 1,853 | 378 | 13,933 |
|  | % | 31.6 | 45.41 | 4.47 | 2.5 | 13.3 | 2.71 | 100 |
| Pakistani/Bangladeshi | Count | 3,976 | 7,271 | 2,526 | 1,918 | 2,404 | 526 | 18,621 |
|  | % | 21.35 | 39.05 | 13.57 | 10.3 | 12.91 | 2.82 | 100 |
| Black/African/Caribbean | Count | 1,464 | 4,502 | 4,399 | 2,903 | 2,497 | 359 | 16,124 |
|  | % | 9.08 | 27.92 | 27.28 | 18 | 15.49 | 2.23 | 100 |
| mixed ethnicities | Count | 993 | 2,418 | 943 | 977 | 1,157 | 133 | 6,621 |
|  | % | 15 | 36.52 | 14.24 | 14.76 | 17.47 | 2.01 | 100 |
| other ethnicities | Count | 4,653 | 7,959 | 2,104 | 1,354 | 3,227 | 326 | 19,623 |
|  | % | 23.71 | 40.56 | 10.72 | 6.9 | 16.44 | 1.66 | 100 |
| Total | Count | 132,087 | 153,913 | 38,867 | 27,338 | 46,364 | 5,695 | 404,264 |
|  | % | 32.67 | 38.07 | 9.61 | 6.76 | 11.47 | 1.41 | 100 |
| Country of birth (Chi2 test P-value=0.000) | | | | | | | | |
| Born in the UK | Count | 94,280 | 107,367 | 25,312 | 19,115 | 28,493 | 3,729 | 278,296 |
|  | % | 33.88 | 38.58 | 9.1 | 6.87 | 10.24 | 1.34 | 100 |
| Not born in the UK | Count | 11,204 | 19,620 | 7,479 | 4,784 | 11,651 | 1,115 | 55,853 |
|  | % | 20.06 | 35.13 | 13.39 | 8.57 | 20.86 | 2 | 100 |
| Missing information | Count | 26,603 | 26,926 | 6,076 | 3,439 | 6,220 | 851 | 70,115 |
|  | % | 37.94 | 38.4 | 8.67 | 4.9 | 8.87 | 1.21 | 100 |
| Total | Count | 132,087 | 153,913 | 38,867 | 27,338 | 46,364 | 5,695 | 404,264 |
|  | % | 32.67 | 38.07 | 9.61 | 6.76 | 11.47 | 1.41 | 100 |

Supplementary Table 5: Distribution of ethnic groups and country of birth by the type of housing tenure in the “Understanding Society” data using Chi2 test tabulation (N=404,264 observations from 67,982 individuals)

Supplementary Table 6: Description of the area of residence (urban or rural) for ethnic groups and country of birth in the “Understanding Society” data using Chi2 test tabulation (N=404,264 observations from 67,982 individuals)

|  | | Urban area | Rural area | Total |
| --- | --- | --- | --- | --- |
| Ethnicity (Chi2 test P-value=0.000) | | | | |
| British-white | Count | 221,221 | 88,371 | 309,592 |
|  | % | 71.46 | 28.54 | 100 |
| Other-white | Count | 14,071 | 5,679 | 19,750 |
|  | % | 71.25 | 28.75 | 100 |
| Indian | Count | 13,715 | 218 | 13,933 |
|  | % | 98.44 | 1.56 | 100 |
| Pakistani/Bangladeshi | Count | 18,539 | 82 | 18,621 |
|  | % | 99.56 | 0.44 | 100 |
| Black/African/Caribbean | Count | 15,952 | 172 | 16,124 |
|  | % | 98.93 | 1.07 | 100 |
| mixed ethnicities | Count | 6,249 | 372 | 6,621 |
|  | % | 94.38 | 5.62 | 100 |
| other ethnicities | Count | 16,488 | 3,135 | 19,623 |
|  | % | 84.02 | 15.98 | 100 |
| Total | Count | 306,235 | 98,029 | 404,264 |
|  | % | 75.75 | 24.25 | 100 |
| Country of birth (Chi2 test P-value=0.000) | | | | |
| Born in the UK | Count | 207,803 | 70,493 | 278,296 |
|  | % | 74.67 | 25.33 | 100 |
| Not born in the UK | Count | 52,203 | 3,650 | 55,853 |
|  | % | 93.46 | 6.54 | 100 |
| Missing information | Count | 46,229 | 23,886 | 70,115 |
|  | % | 65.93 | 34.07 | 100 |
| Total | Count | 306,235 | 98,029 | 404,264 |
|  | % | 75.75 | 24.25 | 100 |

Supplementary Table 7: For people living in urban areas, description of the air pollution exposures by ethnic groups and country of birth at the local authority level (N=306,235 observations)

|  | British white | Other white | Indian | Pakistani/Bangladeshi | Black/African/Caribbean | Mixed ethnicities | Other ethnicities | Born in UK | Not born in UK |
| --- | --- | --- | --- | --- | --- | --- | --- | --- | --- |
| NO_2_ (µg/m^3^) | 14.094 | 16.998 | 21.742 | 22.510 | 24.495 | 21.345 | 19.306 | 15.326 | 21.650 |
| SO_2_ (µg/m^3^) | 1.848 | 1.843 | 1.914 | 1.984 | 1.744 | 1.786 | 1.789 | 1.869 | 1.800 |
| PM10 (µg/m^3^) | 13.989 | 14.933 | 16.510 | 16.361 | 17.754 | 16.547 | 15.809 | 14.440 | 16.593 |
| PM2.5 (µg/m^3^) | 9.497 | 10.162 | 11.463 | 11.343 | 12.325 | 11.445 | 10.919 | 9.833 | 11.486 |
